# Synthesis, Herbicidal Activity Evaluation, and Molecular Docking of Novel Acylthioureas as AHAS Inhibitors

**DOI:** 10.3390/molecules31183162

**Published:** 2026-09-08

**Authors:** Binbin Jiang, Xiying Chen, Xu He, Yunlong Chai, Yan Wang, Ranhong Li

**Affiliations:** College of Plant Protection, Jilin Agricultural University, Changchun 130118, China

**Keywords:** acylthioureas, acetohydroxyacid synthase, herbicidal activity, molecular docking

## Abstract

Acetohydroxyacid synthase (AHAS, EC 2.2.1.6) is a core enzymatic target in agrochemical research for herbicide development and has been widely investigated in recent decades. To develop novel AHAS-targeted herbicides, twenty-three acylthiourea derivatives were synthesized via fragment recombination and bioisosteric replacement strategies in this work. All target compounds were fully characterized by elemental analysis, mass spectrometry, FTIR spectroscopy, and ^1^H NMR spectroscopy. Dose–response trends from the Petri dish assay at 1, 10, and 100 mg L^−1^ show that root-growth inhibition increased synchronously with concentration. Preliminary bioassays across gradient concentrations (1, 10, 100 mg L^−1^) revealed dose-dependent growth-inhibitory effects of several derivatives against the monocot weed *Digitaria adscendens* and dicot weed *Amaranthus retroflexus*. At 100 mg/L pre-emergence treatment, compounds **4v** (76.48 ± 1.47%), **4m** (69.34 ± 1.62%), and **4l** (67.77 ± 1.87%) exhibited the strongest inhibitory activity, comparable to or exceeding bensulfuron-methyl (70.41 ± 1.21%). All synthesized acylthiourea derivatives exhibited less than 20% growth inhibition toward wheat and soybean, demonstrating acceptable crop selectivity. In vivo enzymatic assays at 100 mg L^−1^ showed that **4l**, **4m**, and **4v** achieved AHAS inhibition rates of 38.25 ± 1.81%, 35.74 ± 1.35%, and 38.75 ± 1.93%, comparable to or marginally exceeding that of bensulfuron-methyl (35.64 ± 1.40%). Molecular docking simulations yielded binding energies of −6.67 kcal mol^−1^ (**4l**), −6.29 kcal mol^−1^ (**4m**), and −6.90 kcal mol^−1^ (**4v**), all more favorable than −5.86 kcal mol^−1^ calculated for bensulfuron-methyl, indicating stronger target-enzyme binding affinity for these three compounds. This research suggests that these acylthiourea derivatives may serve as preliminary lead scaffolds for developing novel AHAS inhibitors via subsequent structural derivatization, pending further dose–response, mechanistic, and field-efficacy validation.

## 1. Introduction

As widely reported, weeds act as one of the major biotic stress factors restricting crop growth, leading to remarkable declines in both crop yield and quality [1,2]. Owing to simple operation procedures, outstanding weed control performance and affordable costs, herbicides remain the dominant tool for weed control [3,4]. Although herbicides deliver enormous benefits to agricultural production, their widespread global overuse has induced weed resistance evolution and exerted obvious negative impacts on ecosystems and human well-being [5,6]. Accordingly, the development of novel herbicides remains critical to guarantee food production and delay or even prevent the emergence of weed resistance.

The pioneering research conducted by G. Levitt in the early 1980s established sulfonylurea (SU) herbicides as a landmark breakthrough in agrochemical science, owing to their ultra-low field application rates and environmentally benign properties [7,8]. More than 30 commercial products of SUs have been developed and widely applied in crop protection [8,9,10]. The primary mode of action of SU herbicides relies on the potent inhibition of acetohydroxyacid synthase (AHAS), alternatively named acetolactate synthase (ALS). AHAS inhibition blocks plant amino-acid metabolism and suppresses plant growth, leading to plant death [11,12]. This makes AHAS one of the most attractive enzymes in designing novel herbicidal and fungicidal lead compounds [13,14]. Multiple crystal structures of plant AHAS and AHAS-inhibitor complexes have been resolved, laying a solid foundation for rational inhibitor design [15,16,17,18,19,20,21,22]. In recent years, fragment-based structural modification has become a mainstream strategy for discovering new AHAS-targeted herbicides. Many analogs derived from sulfonylurea scaffolds have been reported; however, several candidates still suffer from unsatisfactory in vivo potency or limited crop safety [9,23,24,25,26]. Meanwhile, most reported AHAS inhibitors rely on structural modification of existing sulfonylurea scaffolds or sulfonylurea analogs without systematic integration of privileged fragments from other herbicide classes [27,28,29,30,31]. Furthermore, the relationship between hybrid-fragment incorporation and AHAS binding affinity remains underexplored, creating a gap for rational design of novel acylthiourea-based herbicide leads.

**Figure 1:**
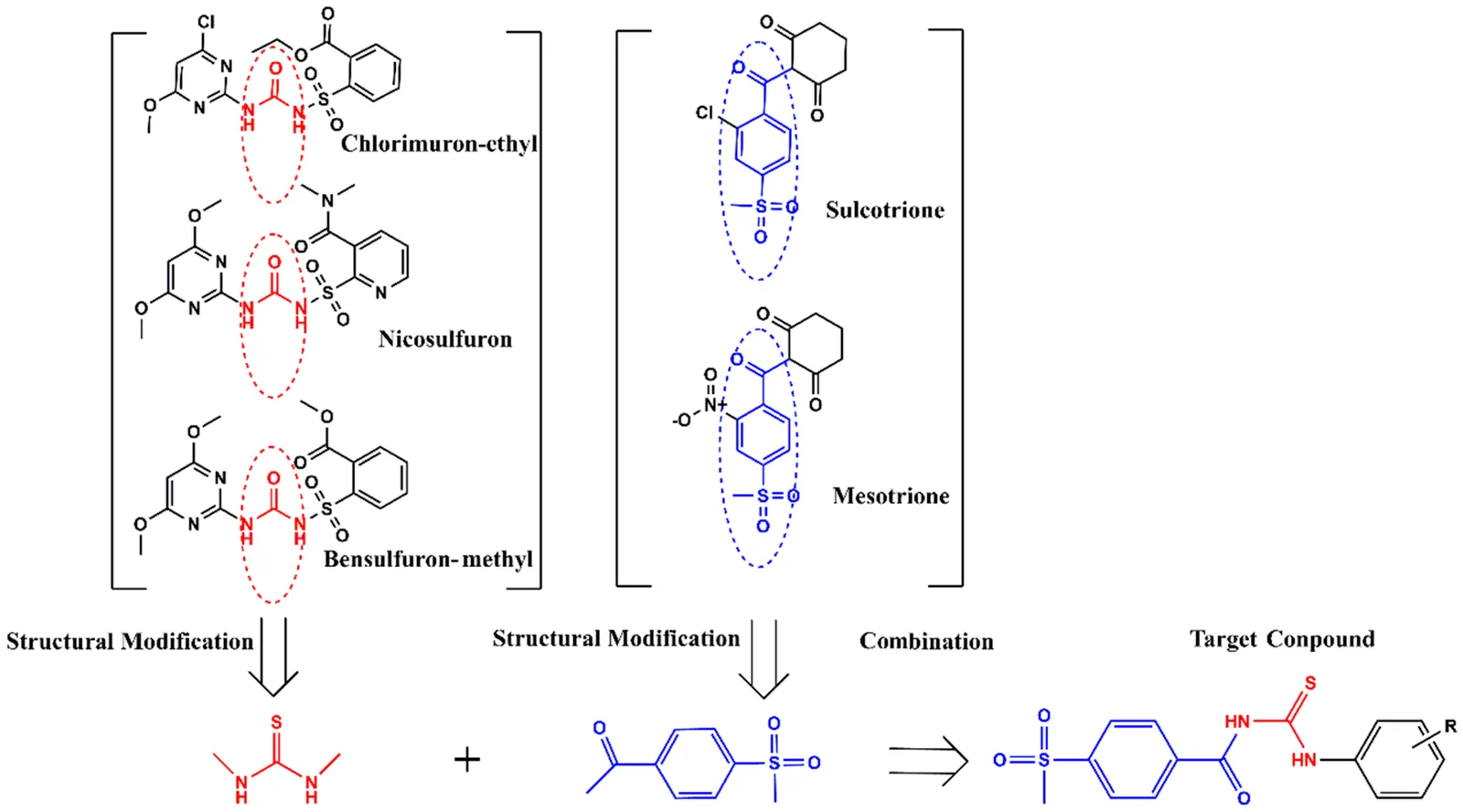
Design strategy of the title molecules.

Since the first acylthiourea derivative was synthesized by M. Nencki in 1873, the acylthiourea skeleton has been widely explored in agrochemical and medicinal chemistry for antifungal, antibacterial, insecticidal, and herbicidal activities [31,32,33,34,35,36,37,38]. Structurally, the carbonyl group within acylthiourea can serve as a bioisostere replacing the sulfonyl moiety in sulfonylurea herbicides. As an extension of our prior research, in which certain acylthiourea analogs exhibited AHAS-inhibitory activity competitive with sulfonylurea herbicides such as bensulfuron-methyl [39,40,41,42], we substituted the sulfonylurea moiety (–SO_2_–) in SUs with the carbonyl group (–C=O–) of acylthiourea via bioisosteric replacement (substitute a functional group with a chemically similar group to preserve or enhance biological activity), and incorporated a mesotrione or sulcotrione-derived aromatic fragment via fragment recombination (assemble bioactive substructures from known pharmacophores to construct new lead compounds). The acylthiourea moiety serves as the core pharmacophore for AHAS inhibition via hydrogen-bond formation in the active-site pocket, while the mesotrione-originated methylsulfonyl-phenyl fragment provides additional bioactivity and favorable crop selectivity, with the goal of enhancing herbicidal activities of the hybrid compounds. Additionally, according to our previous investigations, the molecular size of acylthioureas and substituents on aniline rings can both influence binding affinity toward AHAS. Additionally, our previous investigations demonstrated that both the overall molecular size of acylthiourea skeletons and substituents on aniline rings could affect AHAS binding affinity. Accordingly, in the present work, we retained the methylsulfonylphenyl moiety derived from mesotrione and sulcotrione as a fixed structural building block within the acylthiourea scaffold. Compared with the acylthiourea derivatives reported in our previous studies, these newly designed compounds possessed reduced molecular dimensions, which was expected to enhance their AHAS-binding affinity and herbicidal potency. Furthermore, structural modification of substituents on the aniline ring of the acylthiourea scaffold further regulated the biological performance of the target compounds, thereby facilitating the screening of derivatives with superior herbicidal activity. We hypothesized that this hybrid molecular design could afford compounds with better AHAS-binding affinity and herbicidal potency than conventional acylthiourea–aniline analogs. According to the design strategy (Figure 1), twenty-three previously unreported acylthiourea derivatives were synthesized, and their herbicidal potential was evaluated via bioassays and molecular docking simulations.

## 2. Results and Discussions

### 2.1. Characterization of Acylthiourea Derivatives (**4a**–**4w**)

#### 2.1.1. FTIR

Infrared absorption bands at 3390–3018 cm^−1^, 1697–1650 cm^−1^ and 1269–1205 cm^−1^ were separately assigned to the N–H, C=O and C=S stretching vibrations, verifying the successful incorporation of the acylthiourea functional group within the target molecules. For some of the synthesized compounds, the N–H stretching signals displayed a red shift to lower wavenumbers. This spectral phenomenon originated from intramolecular hydrogen-bonding interactions between carbonyl oxygen and amino hydrogen atoms, a typical feature commonly observed in acylthiourea derivatives [43]. Intense absorption bands at 3088–3002 cm^−1^ verified the existence of aromatic C−H bonds originating from benzene ring stretching vibrations. Medium-intensity bands observed at 1550–1519 cm^−1^ originated from aromatic C–C skeletal vibrations. Furthermore, absorption bands detected at 1157–1141 cm^−1^ were assigned to the C–N bond. All the feature absorption bands were consistent with the data documented in previous reports [44].

#### 2.1.2. ^1^H NMR

All target compounds displayed expected splitting patterns and chemical shifts in their ^1^H NMR spectra. The singlet proton resonance assigned to the –CONH– moiety was observed at δ 12.03–12.73 ppm, while that corresponding to the –CSNH– group was located at δ 11.60–12.29 ppm. These two proton signals were the most useful signals in the ^1^H NMR spectra for confirming the acylthiourea structure of all derivatives [45,46]. The singlet of the thiourea –CSNH– proton displayed a higher chemical shift value than that of the amide –CONH– proton, a phenomenon attributed to intramolecular hydrogen-bonding interactions [47]. The methyl protons of the methylsulfone group produced singlet signals at δ 3.31–3.33 ppm, and aromatic protons of the benzene rings produced a series of multiplet resonances at δ 6.95–8.77 ppm.

Detailed characterization data as well as FTIR and H^1^ NMR spectra for all title compounds (**4a**–**4w**) are provided in the Supplementary Materials.

### 2.2. Herbicidal Activity

Preliminary evaluation of the herbicidal efficacy of all synthesized derivatives was conducted via a Petri dish assay, with *Brassica napus* L. selected as the test plant. As a widely applied sulfonylureas herbicide, bensulfuron-methyl was chosen as positive control. As summarized in Table 1, the root-growth-inhibitory activity of the title compounds increased synchronously with treatment concentration. Furthermore, all the title compounds (except **4h**) achieved over 40% root-growth inhibition at 100 mg L^−1^. Compounds **4l**, **4m**, and **4t** displayed herbicidal efficacy equivalent to bensulfuron-methyl, whereas **4v** exhibited superior herbicidal efficacy relative to the commercial AHAS inhibitor at 100 mg L^−1^.

**Figure 2:**
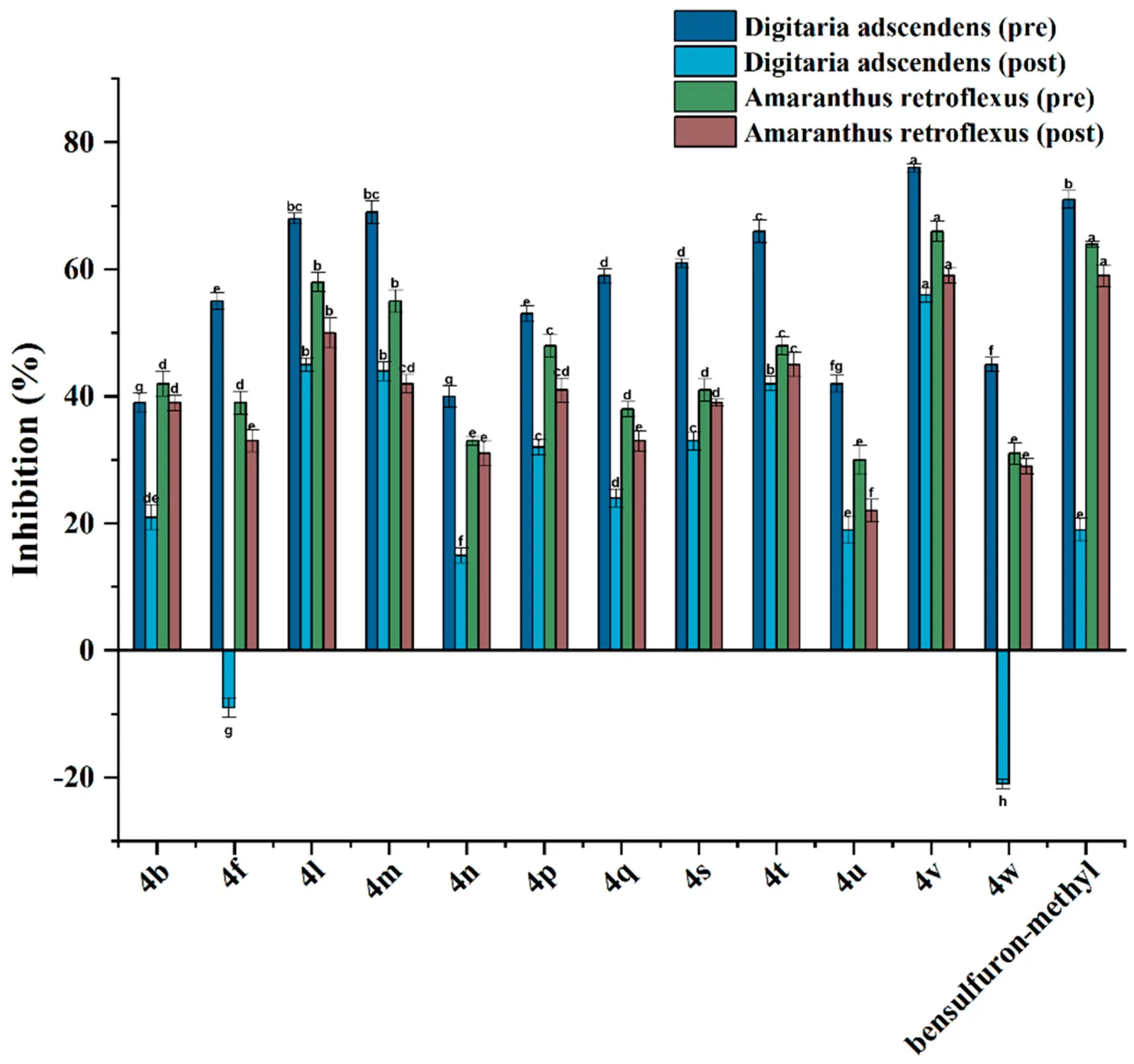
Herbicidal activities of 12 target compounds against *Digitaria adscendens* and *Amaranthus retroflexus* under pre−and post−emergence treatments with 100 mg L^−1^ treatment concentration. Error bars denoted the standard deviation derived from three independent replicates. Statistical significance was determined by one−way ANOVA followed by Tukey’s HSD test (*p* < 0.05) and indicated with different lowercase letters.

Based on the Petri dish screening results, twelve derivatives with relatively strong herbicidal activity were selected for greenhouse pot trials to further evaluate their practical weed control potential. As shown in Figure 2, all test compounds exhibited pre-emergence herbicidal inhibitory activity against *Digitaria adscendens*, a typical monocotyledonous weed. At 100 mg L^−1^ pre-emergence application, the positive control bensulfuron-methyl yielded a 70.41 ± 1.21% weed control rate; by comparison, compounds **4l** (67.77 ± 1.87%), **4m** (69.34 ± 1.62%), and **4t** (65.76 ± 1.53%) exhibited comparable activity, while compound **4v** (76.48 ± 1.47%) displayed statistically significantly higher inhibitory effect on weed growth. For post-emergence application, most compounds (except **4v**) failed to provide effective control on *D. adscendens*, and compounds **4f** and **4w** even showed growth-promoting effects on the tested weeds. Regarding the dicotyledonous weed *A. retroflexus*, only compounds **4l**, **4m** and **4v** displayed acceptable pre-emergence herbicidal efficacy, while other test compounds showed weak weed control efficacy. Post-emergence treatment results for *A. retroflexus* were similar to those observed for *D. adscendens*. Only compound **4v** maintained favorable herbicidal activity. Collectively, these observations indicated that the target compounds exhibited greater herbicidal performance under pre-emergence application at 100 mg L^−1^. Among the tested derivatives, compounds **4l**, **4m**, **4t**, and **4v** showed encouraging weed-suppression potential under these assay conditions.

Structure–activity relationship studies indicated that the substituents of aniline exert prominent effects on the bioactivity of the title compounds. At 100 mg L^−1^ pre-emergence application, we found most of the title compounds with monosubstituted anilines, such as **4m** (R=4-NO_2_), **4q** (R=4-Cl), **4s** (R=4-CH_3_), and **4t** (R=3-CH_3_), showed relatively higher herbicidal activity than those with di- or trisubstituted anilines (with compound **4l** as an exception). This was consistent with our previous work [40], and comparable structure-dependent activity trends have also been reported for commercial sulfonylurea herbicides. The reason for this might be that substituents with smaller spatial volume allow the ligand to fit more easily into the enzyme active pocket. This phenomenon could be utilized for structural design of novel acylthioureas in subsequent research. Moreover, the substitution site on the aniline ring is another critical factor modulating compound bioactivity. Within the dichlorophenyl-substituted series, the 2,6-dichloro derivative (compound **4l**) displayed higher herbicidal activity against weeds compared with its three isomers (**4j**, **4r**, **4n**), which might be attributed to the favorable spatial conformation of 2,6-disubstituted phenyl groups when bound to the enzyme. Notably, compound **4v** showed the highest overall herbicidal activity of all derivatives, and its aniline moiety carries no substituents. This may stem from the compact overall molecular size of compound **4v**, which facilitates the molecule to approach and interact with the target enzyme. With respect to electronic effects, electron-withdrawing substituents (–NO_2_, halogens) on the aniline ring generally correlated with improved biological potency. For example, **4m** (4-NO_2_) and **4q** (4-Cl) outperformed alkyl-substituted analogs such as **4h** (2-CH_3_) at 100 mg L^−1^. The electron-withdrawing groups may enhance the hydrogen-bond acceptor capability of the aniline nitrogen, strengthening ligand–protein interactions. The 3-NO_2_ derivative (**4i**) was less active than the 4-NO_2_ isomer (**4m**), indicating that substitution position modulates the electronic effect on bioactivity. Regarding the correlation between docking scores and bioassay results, a general positive trend was observed: compounds with more negative binding energies (**4v**: −6.90, **4l**: −6.67, **4m**: −6.29 kcal mol^−1^) generally displayed stronger AHAS-inhibitory and herbicidal activity. However, compound **4s** represents a notable outlier, exhibiting a favorable docking score (−6.20 kcal mol^−1^) but relatively weak in vivo AHAS-inhibitory activity. This discrepancy highlights that in silico docking predictions require experimental biological validation, as factors such as cellular uptake, metabolic stability, and in planta bioavailability are not captured by docking simulations.

**Figure 3:**
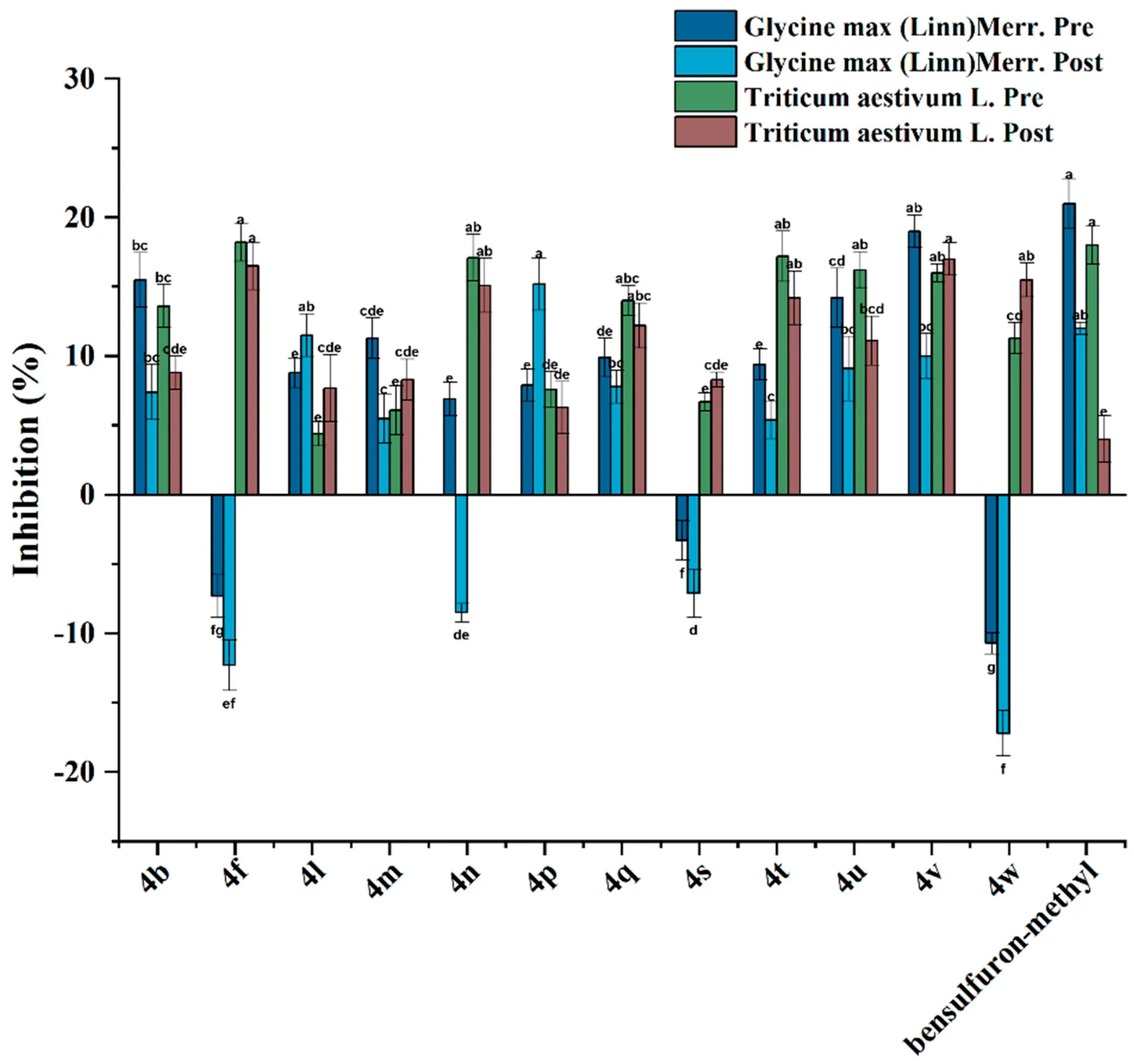
The inhibition rate of 12 target compounds against two crops under pre−and post−emergence conditions with 100 mg L^−1^ treatment concentration. Error bars denoted the standard deviation derived from three independent replicates. Statistical significance was determined by one-way ANOVA followed by Tukey’s HSD test (*p* < 0.05) and indicated with different lowercase letters.

We also conducted crop safety experiments on wheat (*Triticum aestivum* L.) and soybean (*Glycine max (Linn.) Merr.*) to evaluate the influence of the twelve derivatives on crops. As summarized in Figure 3, all compounds induced less than 20% growth inhibition rate for both gramineous crop and dicotyledonous crop. Interestingly, compounds **4f**, **4n**, **4s**, **4w** showed certain promotive effects on soybean; compound **4w** even produced up to 18.47 ± 1.65% growth promotion in soybean seedlings. These results indicate that the synthesized acylthiourea derivatives exhibited preliminary crop selectivity at 100 mg L^−1^, with all compounds producing less than 20% growth inhibition on both wheat and soybean. Comprehensive safety evaluation across gradient concentrations, including determination of selectivity indices, will be necessary to fully assess their field applicability.

### 2.3. AHAS Activity in Plant Extracts Following In Vivo Compound Treatment

**Figure 4:**
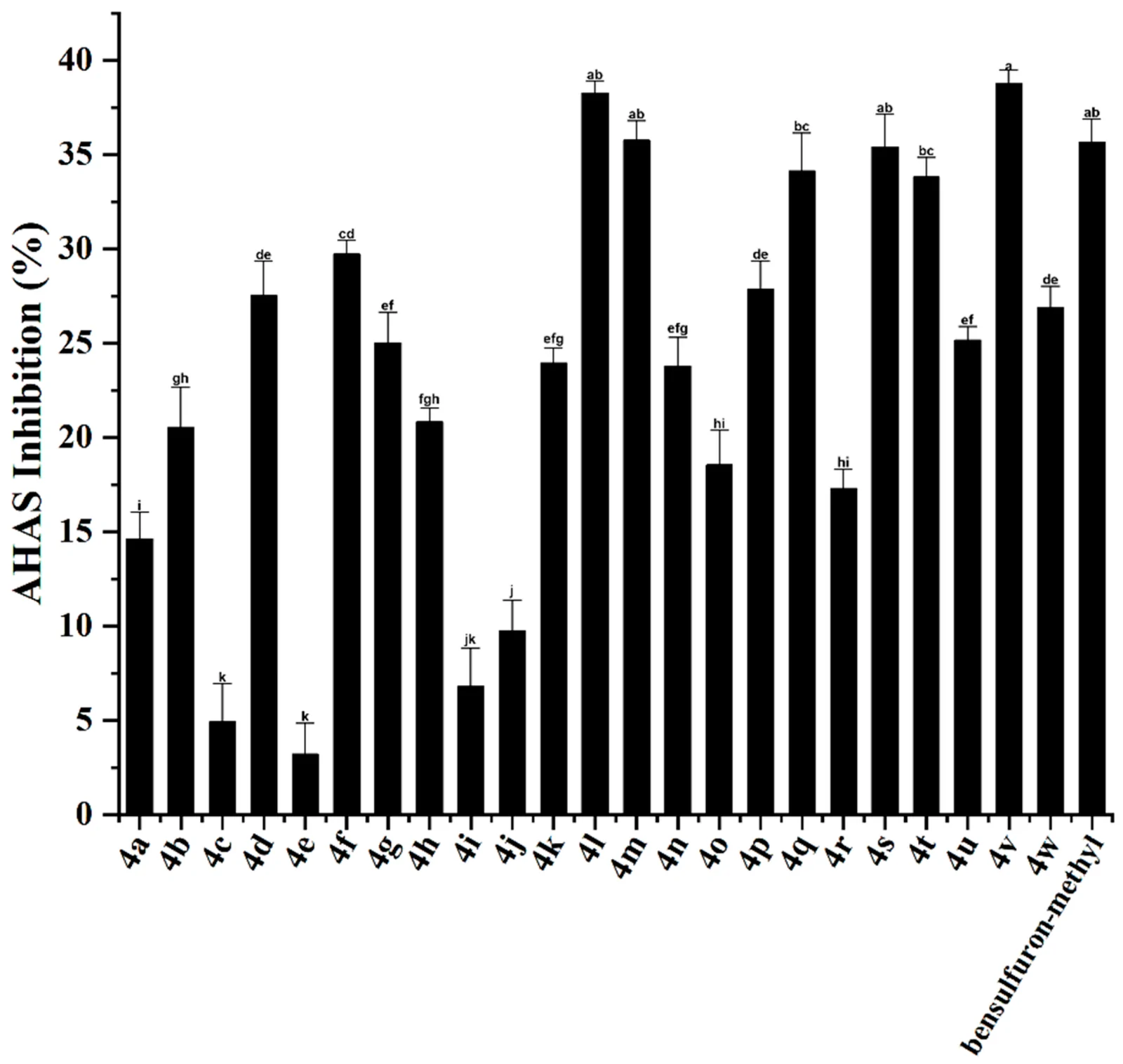
Inhibitory activities of 12 compounds against AHAS in vivo at 100 mg L^−1^. Error bars representd the standard deviation of three replicates. Statistical significance was determined by one-way ANOVA followed by Tukey’s HSD test (*p* < 0.05) and indicated with different lowercase letters.

AHAS was chosen as the target enzyme to evaluate AHAS-inhibitory potency of the title compounds, owing to their structural homology and comparable herbicidal performance relative to bensulfuron-methyl. Figure 4 summarizes the in vivo enzymatic test data acquired at 100 mg L^−1^, which demonstrate that all target compounds exhibited AHAS-inhibitory activity. Among all title derivatives, compounds **4l** (38.25 ± 1.81%), **4m** (35.74 ± 1.35%), **4s** (35.23 ± 2.15%) and **4v** (38.75 ± 1.93%) exhibited AHAS inhibition rates comparable to or numerically higher than bensulfuron-methyl (35.64 ± 1.40%) at 100 mg L^−1^. Moreover, the results of the in vivo AHAS activity test were roughly similar to those of the herbicidal activity test. As bensulfuron-methyl serves as a commercial AHAS-inhibiting herbicide, these findings imply that the synthesized derivatives may produce their herbicidal activity partially via AHAS inhibition. Nevertheless, further mechanistic investigations are still required to fully validate this proposed mode of action.

### 2.4. Molecular Docking

**Figure 5:**
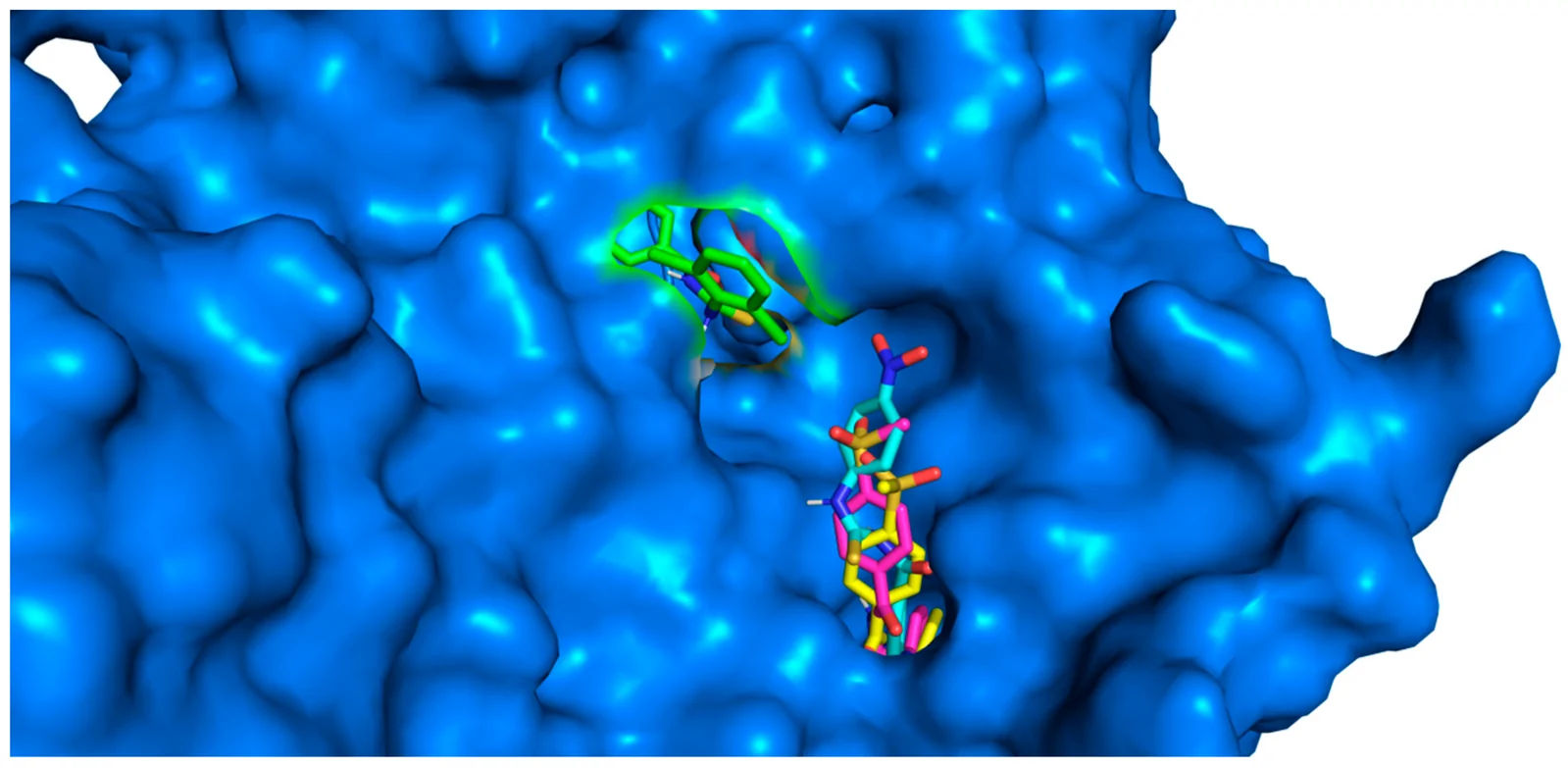
Molecular docking simulation of title compounds in the active cavity of AHAS.

Molecular docking is known as a powerful in silico approach to explore ligand–enzyme intermolecular interactions, and this method has been widely adopted to guide the rational design of novel compounds [48,49]. Accordingly, we conducted molecular docking simulations between the AHAS protein and all target derivatives to elucidate the potential molecular mechanism responsible for their bioactivity. The co-crystal structure of *Arabidopsis thaliana* AHAS bound to chlorsulfuron (PDB ID: 1YHZ) was selected as the molecular docking template. This structure has been extensively adopted for computational research on AHAS-directed herbicide inhibitors, which enables straightforward comparison against earlier docking outputs. As shown in Table 2, the title compounds were ranked according to their binding energies after 50 times independent docking between the target proteins and each ligand. Compared to bensulfuron-methyl (−5.86 kcal mol^−1^), compounds **4l**, **4m**, **4s** and **4v** exhibited more favorable binding energies of −6.67, −6.29, −6.20, and −6.90 kcal mol^−1^, respectively. This indicated that these four compounds had favorable binding affinity for AHAS, which aligned with the results of in vivo AHAS inhibitory tests. Superimposition of the lowest-energy optimal docking conformations was carried out for all title derivatives. Figure 5 illustrates each acylthiourea ligand embedded within the catalytic active pocket of the AHAS subunit. This binding mode is consistent with the docking results of sulfonylurea derivatives reported in the previous literature and our prior research [39,40,41,50]. Therefore, we infer that these acylthiourea derivatives represent a novel class of AHAS-inhibiting herbicides. Notably, although compound **4s** exhibited a favorable docking binding energy, it showed relatively weak AHAS-inhibitory activity in enzymatic assays and low practical weed-control potential in greenhouse pot trials when compared with compounds **4l**, **4m**, and **4v**. Therefore, it was excluded from further detailed interaction analyses. Only compounds **4l**, **4m** and **4v**, which performed well in both docking simulations and enzymatic inhibition experiments, were selected for detailed intermolecular interaction analysis.

**Figure 6:**
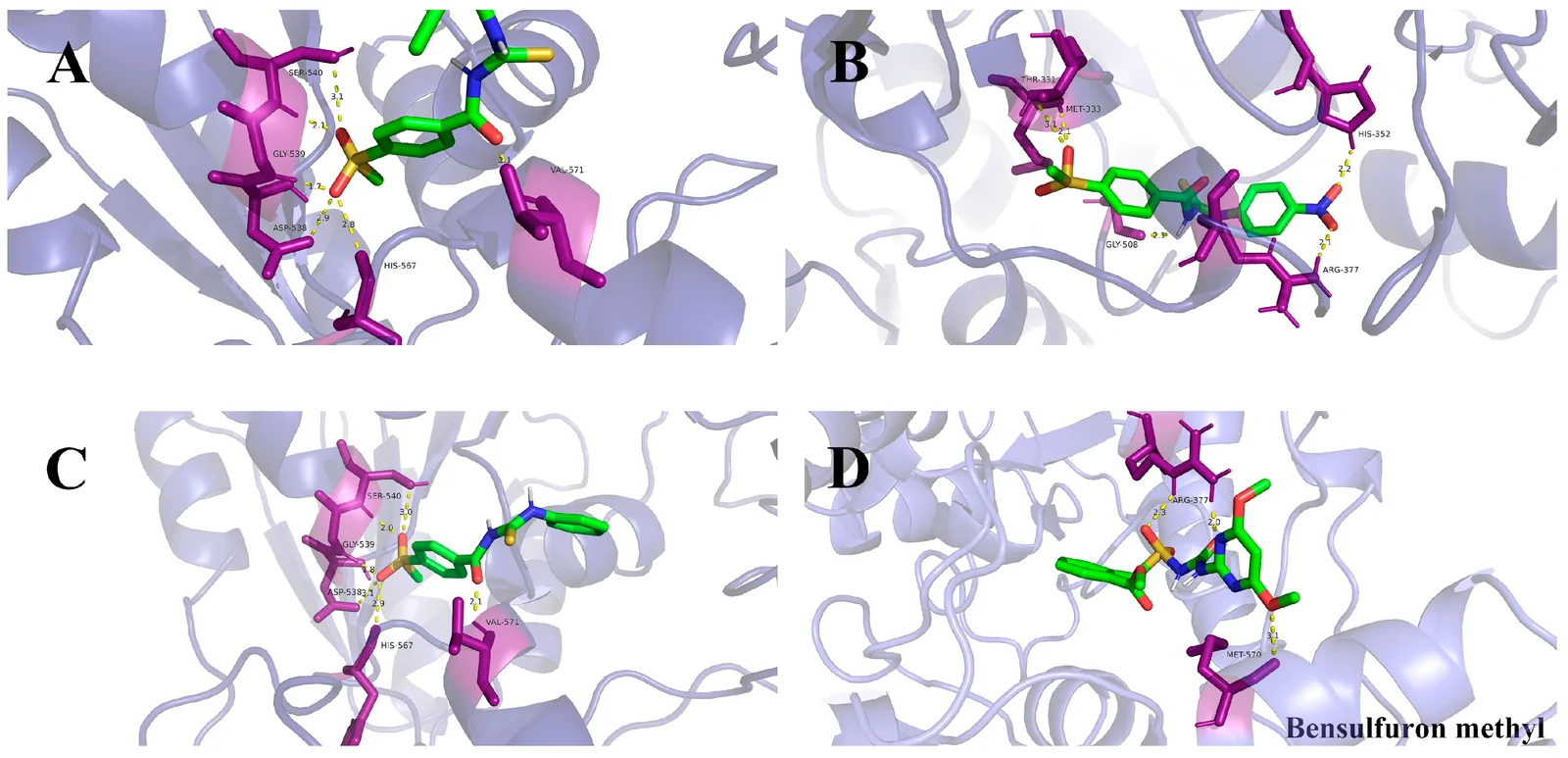
Binding mode of compounds. (**A**) Binding mode of compound **4l**; (**B**) Binding mode of compound **4m**; (**C**) Binding mode of compound **4v**; (**D**) Binding mode of bensulfuron-methyl.

As illustrated in Figure 6A, six intermolecular hydrogen bonds formed between compound **4l** and the target AHAS protein. Five of these hydrogen bonds originate from the two oxygen atoms of the methylsulfonyl substituent, which interact with four amino acid residues: Ser540, Gly539, Asp538, and His567, and a sixth hydrogen bond connects the acyl oxygen on the acylthiourea moiety to Val571. For compound **4m** (Figure 6B), five hydrogen bonds exist between the ligand and AHAS. Its methylsulfonyl group establishes two hydrogen-bond contacts with Met333 and Thr331. The NH group of the acylthiourea moiety forms one hydrogen bond with Gly508, while the nitro substituent on the aniline ring generates two hydrogen bonds with His352 and Arg377. As shown in Figure 6C, the binding mode of compound **4v** resembles that of **4l**. The methylsulfonyl group of **4v** creates five hydrogen bonds with Ser540/Gly539/Asp538/His567, with one supplementary hydrogen bond bridging the acyl group and Val571. The superior inhibitory activity of **4l** and **4v** suggests that these five residues (Ser540/Gly539/Asp538/His567/Val571) may serve as important interaction partners for acylthiourea ligands in simulated docking poses. Site-directed mutagenesis studies would be required to experimentally verify their functional importance. As shown in Figure 6D, the positive control bensulfuron-methyl only forms three hydrogen bonds with AHAS: two from its sulfonylurea moiety to Arg377, and one from pyrimidine methoxy to Met570. Compared with bensulfuron-methyl (only three hydrogen bonds with Arg377 and Met570), **4l**, **4m**, and **4v** establish five-to-six hydrogen-bond interactions within the AHAS binding cavity. The increased number of hydrogen-bond contacts partially accounts for their more favorable binding energies and stronger in vivo AHAS-inhibitory performance. Specifically, the methylsulfonyl group in **4l** and **4v** engages a cluster of four residues (Ser540, Gly539, Asp538, His567) that bensulfuron-methyl does not contact, anchoring the ligand more firmly in the active pocket. For 4m, the nitro substituent on the aniline ring provides additional hydrogen-bond donor/acceptor interactions with His352 and Arg377, compensating for the absence of the 2,6-dichloro substitution pattern. This observation indicates that strengthened hydrogen-bonding contacts between the ligand and protein pocket facilitate enhanced compound bioactivity. Hence, rational structural optimization to strengthen hydrogen-bond interactions between acylthiourea derivatives and AHAS represents an effective strategy to discover new AHAS-inhibiting herbicides. It should be noted that molecular docking provides in silico supportive evidence for ligand–protein interactions rather than definitive experimental proof. The observed binding modes and interaction patterns should be interpreted as hypotheses that warrant further experimental validation, such as site-directed mutagenesis or co-crystallization studies.

## 3. Materials and Methods

### 3.1. Instruments and Chemicals

A micro melting-point apparatus (Tech. Instrument Co., Ltd., Beijing, China) was utilized to determine melting points of all title compounds. A Bruker AVANCE-400 spectrometer (Bruker, Bremen, Germany) operating at 400 MHz was employed to collect the ^1^H NMR spectra of synthesized compounds. DMSO-d_6_ served as the dissolving solvent, and tetramethylsilane (TMS) was adopted as the internal standard. A PerkinElmer Vario EL III elemental analyzer (Perkin Elmer, Waltham, MA, USA) was used to perform elemental microanalysis. A Shimadzu FTIR-8400S spectrophotometer (SHIMADZU, Kyoto, Japan) was utilized to record FTIR spectra over the wavenumber range of 400–4000 cm^−1^. Thin-layer chromatography (TLC) was applied to track the progress of all synthetic transformations. Mass spectra were acquired using an Agilent 1260-6460 LC–MS system (Agilent, Santa Clara, CA, USA). A Rayto RT-6100 microplate reader (Rayto Life and Analytical Science Co., Ltd., Shenzhen, China) was used to quantify signals for enzyme activity assays.

### 3.2. Experimental

#### General Procedure for the Synthesis of Acylthiourea Derivatives

**Scheme 1:**
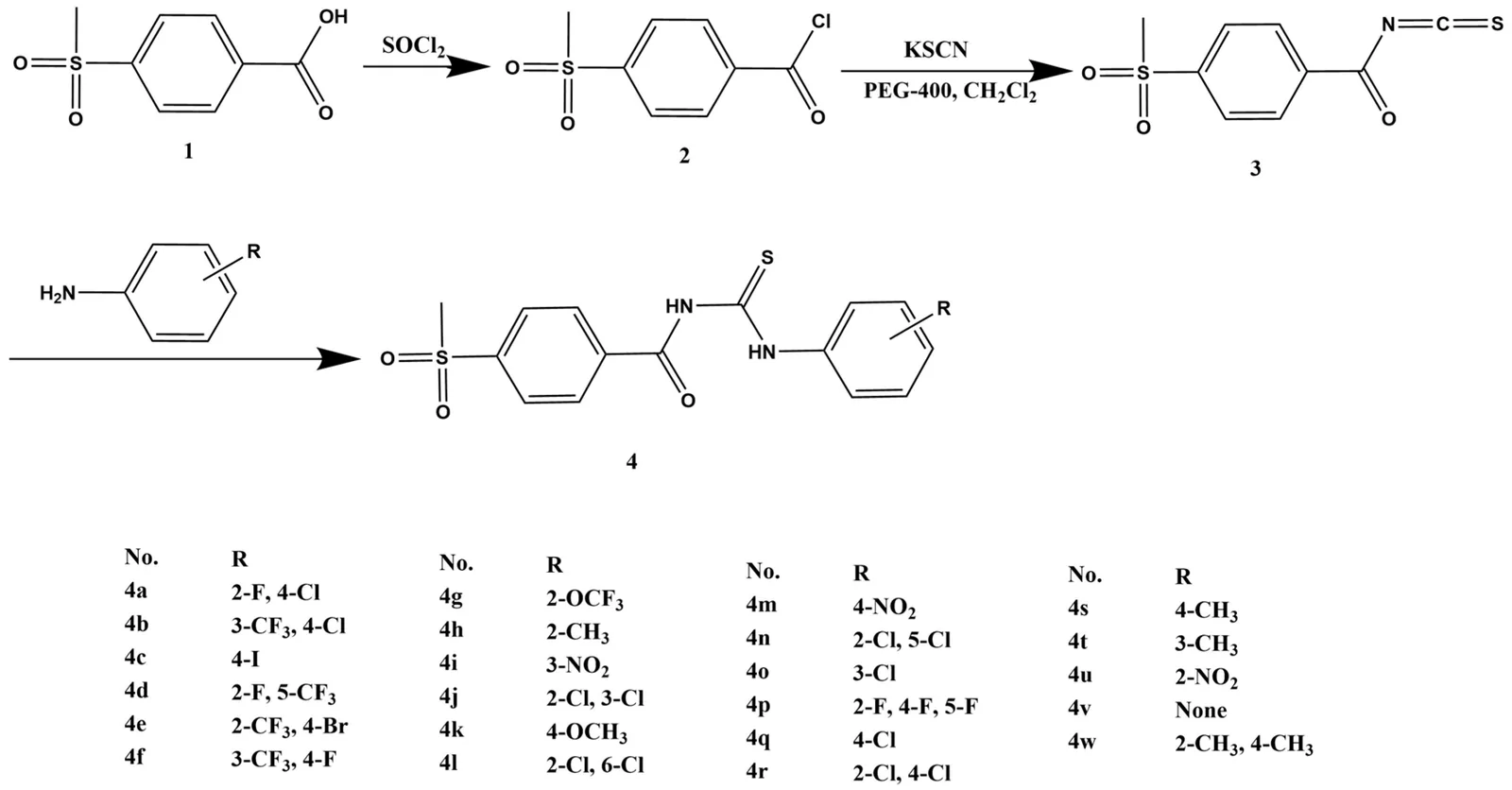
Synthesis route of the title compounds.

The synthetic route for all target derivatives was designed based on our previously reported procedure [40]. As shown in Scheme 1, the synthetic procedure is described as follows: 15 mmol 4-methylsulfonyl benzoic acid (intermediate **1**) and 15 mL of thionyl chloride were loaded into a round-bottomed flask. After 4 h of reflux treatment, the reaction system was concentrated to obtain the intermediate **2**. Subsequently, potassium thiocyanate (15 mmol), catalyst (PEG-400, 3 mol % relative to potassium thiocyanate), and an appropriate volume of anhydrous CH_2_Cl_2_ were combined with intermediate **2** to form a suspension. The suspension was stirred for 2 h, and then filtered to obtain intermediate **3**. Next, 10 mmol of the corresponding substituted aniline was added to intermediate **3**. After gentle stirring for 1 h, the mixture was filtered, and ethanol was used to rinse the filter residues. Finally, the pure target compounds were obtained through recrystallization from a ternary mixed solvent of DMF–EtOH–H_2_O.

### 3.3. Herbicidal Activity Assay

#### 3.3.1. Petri Dish Test

Seeds of *Brassica napus* L. were chosen as test plants for preliminary screening of the biological activities of all title compounds through a Petri dish test. Stock working solutions were formulated by mixing 10 mg of each test compound with 0.05 mL Tween 80 (emulsifier) and 0.25 mL DMF (solvent), followed by serial dilution with deionized water to final concentrations of 1, 10, and 100 mg L^−1^. Parallel treatments containing bensulfuron-methyl were set up as the positive control under matching solvent and culture conditions, while blank controls were prepared without any test compound addition.

Prior to treatment, *B. napus* seeds were pre-incubated at 30 ± 1 °C in darkness for 24 h to complete germination. Twenty uniform pre-germinated seeds were transferred to each Petri dish and overlaid with filter paper soaked in 5 mL test solution. All dishes were incubated at 30 ± 1 °C under continuous dark conditions for 48 h. Each experiment was repeated in triplicate. The root-growth inhibition efficacy of each compound was quantified via Equation (1):

(1) $$\text{Control effect}\left(\%\right)=\frac{L_{b}-L_{t}}{L_{b}}\times 100\%$$

where *L_t_* and *L_b_* represented the mean root lengths of *Brassica napus* L. seedlings under treatment with test compounds and blank control groups, respectively.

#### 3.3.2. Greenhouse Herbicidal Activity Test

Greenhouse pot trials were conducted against *Digitaria adscendens* and *Amaranthus retroflexus* to assess the herbicidal potency of all title compounds. Pots with an 8 cm inner diameter were filled with 100 g soil (60 g sand and 40 g clay). Twenty pre-germinated seeds were sown at a depth of 0.6 cm inside each pot and cultivated under controlled greenhouse conditions (25 °C, 60–70% relative humidity, 14 h light/10 h dark photoperiod). Each treatment was performed with 3 biological replicates (three pots per replicate, 20 seeds per pot). Pots were arranged according to a completely randomized layout and weekly re-randomization was carried out to mitigate location-dependent experimental artifacts. Stock solutions were formulated by dissolving each compound in 2.5 mL DMF supplemented with 0.5 mL Tween 80, followed by serial dilution with deionized water to obtain the test solution with required concentration. Positive controls were prepared with bensulfuron-methyl under identical solvent conditions, while blank controls contained only DMF and Tween 80 without any herbicidal compounds. For pre-emergence treatment, 5 mL test solution of each title compound was sprayed on the soil after 24 h seed incubation. For post-emergence treatment, 5 mL test solution was sprayed onto weeds at the two-leaf growth stage. After 20 days of greenhouse cultivation, surviving weed individuals per pot were counted, and weed control efficacy was calculated via Equation (2):

(2) $$\text{Control effect}\left(\%\right)=\frac{N_{b}-N_{t}}{N_{b}}\times 100\%$$

where *N_t_* and *N_b_* were the numbers of surviving weeds per pot under test compound treatments and blank control treatments, respectively.

#### 3.3.3. AHAS Activity in Plant Extracts Following In Vivo Compound Treatment

Seed incubation of *Brassica napus* L. was performed at 30 ± 1 °C under dark conditions for 48 h using the established Petri dish assay protocol. Pre-germinated seeds (0.5 g) were ground using phosphate-buffered saline (PBS; 50 mmol L^−1^, pH 7.0) for sample preparation. The obtained homogenate underwent centrifugation at 4000 r·min^−1^ and 4 °C over a 15-min period, and the recovered supernatant served as crude enzyme extract. A commercially available ELISA kit was used to measure AHAS activity at 450 nm according to the guidelines of the manufacturer. Bensulfuron-methyl was included as positive control. Each enzyme assay was carried out in triplicate using separate biological samples. All test solutions were prepared following the protocol outlined in Section 3.3.2. The relative AHAS inhibition rate was calculated as:

(3) $$\text{Inhibition}\left(\%\right)=\frac{1-{OD}_{treatment}}{{OD}_{control}}\times 100\%$$

where *OD_treatment_* and *OD_control_* represent the absorbance values of compound-treated and solvent-treated samples, respectively. Bensulfuron-methyl was included as positive control. Each assay was carried out in triplicate.

#### 3.3.4. Crop Safety Test

Wheat (*Triticum aestivum*, gramineous crop) and soybean (*Glycine max (Linn.) Merr.*, dicotyledonous crop) were chosen for crop safety evaluation. All cultivation and spraying operations strictly complied with the experimental procedures detailed in Section 3.3.2, Greenhouse herbicidal activity test.

### 3.4. Molecular Docking

All target acylthiourea derivatives were sketched as 2D structures in ChemDraw ultra 18.0, and their conformations were subjected to MM2 force field energy minimization within Chem3D 18.0. Fully optimized 3D ligand molecules (**4a**–**4w**) were saved as input files for subsequent in silico docking simulations. The crystal structure of plant AHAS was retrieved from the PDB database (PDB accession code: 1YHZ). Prior to docking, co-crystallized ligands, water molecules, and metal ions were removed from the PDB 1YHZ structure to obtain the apo-protein conformation via PyMOL 3.1. This pre-processing step allows uniform docking of all test ligands into the unoccupied inhibitor-binding pocket of AHAS, eliminating interference from pre-bound co-crystallized small molecules.

The optimized ligand molecules were docked to AHAS via Auto Dock 4.2.6 software, and a total of 50 separate docking runs were conducted with the Genetic Algorithm (GA) computational module. Most of the parameters for the docking calculation were set to the default values recommended by the software. The lowest-energy binding pose of each compound was isolated for systematic characterization of protein–ligand intermolecular contacts. The pose with the minimum binding free energy was selected for visual analysis of ligand–protein binding modes using PyMOL 3.1. For comparison, the commercial inhibitor bensulfuron-methyl was processed and docked following the exact same computational workflow as all test ligands. We performed re-docking of the native co-crystallized chlorsulfuron ligand into the AHAS to assess the performance of the docking protocol. The re-docked pose showed good overlap with the original crystal conformation (RMSD < 2.0 Å), confirming the reliability of our docking parameter setup.

### 3.5. Statistical Analysis

Every biological experiment was conducted with three separate biological replicates, and experimental outcomes are reported as the mean ± standard deviation (SD). A one-way analysis of variance (ANOVA) coupled with Tukey’s honestly significant difference (HSD) post hoc procedure was applied for inter-group comparisons. Statistical differences between treatments were recognized as significant when *p* < 0.05. All statistical computations were completed with SPSS software (version 30.0).

## 4. Conclusions

Herein, we designed and synthesized a series of twenty-three novel acylthiourea derivatives via bioisosteric replacement and fragment recombination, incorporating a mesotrione-derived aromatic fragment into the acylthiourea scaffold. At 100 mg L^−1^ treatment concentration, compounds **4l**, **4m**, and **4v** exhibited herbicidal activity comparable to bensulfuron-methyl, with all compounds producing less than 20% growth inhibition on wheat and soybean, indicating preliminary crop selectivity. In vivo AHAS activity assays showed that **4l**, **4m**, and **4v** achieved AHAS inhibition rates comparable to or numerically higher than bensulfuron-methyl at the tested concentration. Molecular docking simulations provided supportive in silico evidence that these three compounds form more hydrogen-bond interactions with AHAS active-site residues than bensulfuron-methyl, consistent with their more favorable binding energies. The present findings are preliminary and imply that the synthesized acylthiourea analogs could act as lead scaffolds for follow-up structural modification. These compounds can also offer valuable clues to design new AHAS-targeting inhibitors via further structural derivatization. However, systematic investigations including full dose–response determination, in vitro AHAS kinetic assays, deeper mechanistic studies, field-efficacy trials, and comprehensive toxicological and environmental-safety assessments are required before their practical herbicidal potential can be fully validated.

## Figures and Tables

**Table 1:** The inhibitory effects of title compounds (**4a**–**4w**) on root growth of *Brassica napus* L.

Compound	R	Inhibition (%)		
		1 mg L^−1^	10 mg L^−1^	100 mg L^−1^
**4a**	2-F, 4-Cl	19.84 ± 1.23 j	25.00 ± 1.83 lm	50.65 ± 1.59 hi
**4b**	3-CF_3_, 4-Cl	58.87 ± 1.01 b	63.71 ± 1.54 bc	63.86 ± 1.90 f
**4c**	4-I	31.61 ± 1.3 gh	47.66 ± 1.78 ghi	48.48 ± 1.48 ij
**4d**	2-F, 5-CF_3_	40.48 ± 1.12 e	44.35 ± 0.67 ij	54.03 ± 1.26 gh
**4e**	2-CF_3_, 4-Br	24.19 ± 2.01 i	34.27 ± 1.87 kL	48.23 ± 1.33 ij
**4f**	3-CF_3_, 4-F	−8.87 ± 1.57 k	35.48 ± 1.18 k	70.13 ± 1.46 cde
**4g**	2-OCF_3_	37.23 ± 1.13 ef	44.06 ± 1.72 ij	55.90 ± 1.21 g
**4h**	2-CH_3_	−7.46 ± 1.21 k	20.97 ± 1.39 m	25.16 ± 1.71 L
**4i**	3-NO_2_	28.55 ± 1.15 h	29.90 ± 2.33 L	44.68 ± 2.41 jk
**4j**	2-Cl, 3-Cl	31.61 ± 2.13 gh	33.06 ± 1.61 kL	42.46 ± 1.49 k
**4k**	4-OCH_3_	33.23 ± 1.54 fg	45.56 ± 1.97 hij	51.01 ± 1.94 ghi
**4l**	2-Cl, 6-Cl	47.98 ± 1.34 c	64.92 ± 1.52 b	77.98 ± 1.87 ab
**4m**	4-NO_2_	45.03 ± 0.87 cd	57.66 ± 1.06 de	78.94 ± 1.62 ab
**4n**	2-Cl, 5-Cl	34.68 ± 1.77 fg	53.15 ± 1.46 ef	65.10 ± 0.53 f
**4o**	3-Cl	33.64 ± 1.69 fg	42.94 ± 1.19 ij	49.19 ± 1.93 hij
**4p**	2-F, 4-F, 5-F	20.16 ± 1.27 ij	49.77 ± 1.16 fgh	67.10 ± 1.77 def
**4q**	4-Cl	78.71 ± 1.07 a	43.39 ± 1.38 ij	68.13 ± 1.18 def
**4r**	2-Cl, 4-Cl	41.13 ± 0.65 de	42.77 ± 1.41 j	44.35 ± 1.22 jk
**4s**	4-CH_3_	56.45 ± 1.82 b	52.82 ± 1.15 ef	71.69 ± 1.68 cd
**4t**	3-CH_3_	35.48 ± 1.33 fg	74.27 ± 2.14 a	74.56 ± 1.64 bc
**4u**	2-NO_2_	−6.45 ± 0.66 k	64.92 ± 1.17 b	65.29 ± 0.44 ef
**4v**	None	35.32 ± 1.11 fg	50.87 ± 0.76 fg	81.77 ± 1.47 a
**4w**	2-CH_3_, 4-CH_3_	21.37 ± 1.37 ij	63.71 ± 1.80 bc	64.31 ± 1.83 f
bensulfuron-methyl	-	55.61 ± 1.51 b	59.54 ± 1.86 cd	78.01 ± 1.21 ab

Data represent mean ± standard deviation (*n* = 3). Statistical analysis was performed via one-way ANOVA and subsequent Tukey’s HSD test at a significance level of *p* < 0.05. Statistically different values were indicated with different lowercase letters.

**Table 2:** Docking scores.

Compound	R	Docking Score (kcal mol^−1^)
**4a**	2-F, 4-Cl	−4.13
**4b**	3-CF_3_, 4-Cl	−4.35
**4c**	4-I	−2.89
**4d**	2-F, 5-CF_3_	−4.32
**4e**	2-CF_3_, 4-Br	−2.60
**4f**	3-CF_3_, 4-F	−5.01
**4g**	2-OCF_3_	−4.23
**4h**	2-CH_3_	−4.33
**4i**	3-NO_2_	−3.37
**4j**	2-Cl, 3-Cl	−3.73
**4k**	4-OCH_3_	−4.34
**4l**	2-Cl, 6-Cl	−6.67
**4m**	4-NO_2_	−6.29
**4n**	2-Cl, 5-Cl	−4.41
**4o**	3-Cl	−4.26
**4p**	2-F, 4-F, 5-F	−5.11
**4q**	4-Cl	−5.79
**4r**	2-Cl, 4-Cl	−4.08
**4s**	4-CH_3_	−6.20
**4t**	3-CH_3_	−5.49
**4u**	2-NO_2_	−4.52
**4v**	None	−6.90
**4w**	2-CH_3_, 4-CH_3_	−4.81
bensulfuron-methyl	-	−5.86

## Data Availability

The original contributions presented in this study are included in the article and the Supplementary Materials. Further inquiries may be directed to the corresponding authors.

## Supplementary Materials

The following supporting information can be downloaded at https://www.mdpi.com/article/10.3390/molecules31183162/s1. File S1: 1. Characterization data of acylthiourea derivatives (**4a**–**4w**); 2. FTIR and ^1^H NMR spectra of acylthiourea derivatives (**4a**–**4w**).
