# Supplementary material for: Synthesis, Herbicidal Activity Evaluation, and Molecular Docking of Novel Acylthioureas as AHAS Inhibitors
Source: Molecules. 2026 Sep 8;31(18):3162. doi: 10.3390/molecules31183162 (PMC13609640; doi:10.3390/molecules31183162)

Synthesis, Herbicidal Activity Evaluation, and Molecular Docking of Novel Acylthioureas as AHAS Inhibitors

Binbin Jiang, Xiying Chen, Xu He, Yunlong Chai, Yan Wang and Ranhong Li *

College of Plant Protection, Jilin Agricultural University, Changchun 130118, China

* Correspondence: liranhong@jlau.edu.cn

1. Characterization data of acylthiourea derivatives (4a - 4w)

4a: *N*-((4-chloro-2-fluorophenyl)carbamothioyl)-4-(methylsulfonyl)benzamide

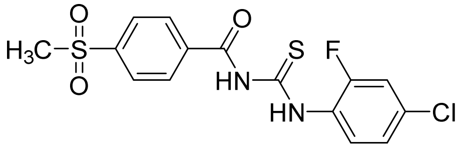

Yield 87%. Light yellow powder. m.p: 208-210°C. Anal. Found, %: C, 46.63; H,3.31; N, 7.11. $C_{15}H_{12}ClFN_2O_3S_2$. Calculated, %: C, 46.57; H,3.13; N, 7.24. FT-IR (KBr) cm^{-1}: 3274, 3091 (N-H), 3082 (=C-H_{Ar}), 1674 (C=O), 1529 (C=C_{Ar}), 1255 (C=S), 1145 (C-N). ^{1}H NMR (400 MHz, DMSO-d_6): δ, ppm 12.31 (s, 1H), 12.15 (s, 1H), 8.22-8.15 (m, 2H), 8.1-8.04 (m, 2H), 7.97 (t, *J* = 8.5 Hz, 1H), 7.61 (dd, *J* = 10.2, 2.3 Hz, 1H), 7.37 (dt, *J* = 8.7, 1.7 Hz, 1H), 3.32 (s, 3H). LC-MS (ESI) *m/z* (*rel*, %): 386.02 $[M + H]^+$ (100).

4b:*N*-((4-chloro-3-(trifluoromethyl)phenyl)carbamothioyl)-4-(methylsulfonyl)benzamide

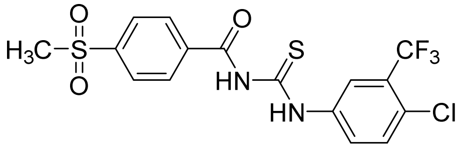

Yield 84%. White crystal. m.p: 189-192°C. Anal. Found, %: C, 44.02; H, 2.56; N, 6.32. $C_{16}H_{12}ClF_3N_2O_3S_2$. Calculated, %: C, 43.99; H, 2.77; N, 6.41. FT-IR (KBr) cm^{-1}: 3365, 3128 (N-H), 3087 (=C-H_{Ar}), 1685 (C=O), 1539 (C=C_{Ar}), 1253 (C=S), 1147 (C-N). LC-MS *m/z* (*rel*, %): 435.99 $[M + H]^+$ (100). ^{1}H NMR (400 MHz, DMSO-d_6) δ 12.03 (s, 1H), 11.61 (s, 1H), 8.37 (d, *J* = 2.4 Hz, 1H), 8.24-8.17 (m, 2H), 8.12 (dq, *J* = 8.9, 2.0 Hz, 3H), 7.79-7.72 (m, 1H), 3.32 (s, 3H).

4c: *N*-((4-iodophenyl)carbamothioyl)-4-(methylsulfonyl)benzamide

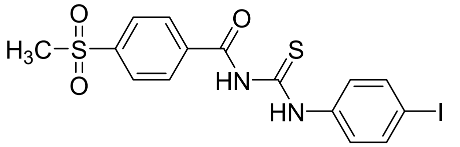

Yield 91%. Light yellow powder. m.p: 280-281°C. Anal. Found, %: C, 39.27; H, 2.81; N, 6.37. $C_{15}H_{13}IN_2O_3S_2$. Calculated, %: C, 39.14; H, 2.85; N, 6.09. FT-IR (KBr) cm^{-1}: 3317, 3168 (N-H), 3070 (=C-H_{Ar}), 1650 (C=O), 1521 (C=C_{Ar}), 1255 (C=S), 1155 (C-N). LC-MS *m*/*z* (*rel*, %): 459.94 $[M + H]^+$ (100). ^{1}H NMR (400 MHz, DMSO-d_6) δ 12.15 (s, 1H), 11.60 (s, 1H), 8.22-8.14 (m, 2H), 8.13-8.07 (m, 2H), 7.78-7.70 (m, 2H), 7.69-7.61 (m, 2H), 3.31 (s, 3H).

4d:*N*-((2-fluoro-5-(trifluoromethyl)phenyl)carbamothioyl)-4-(methylsulfonyl)benzamide

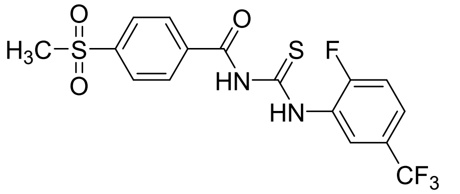

Yield 86%. Light yellow powder. m.p: 202-203.5°C. Anal. Found, %: C, 45.83; H, 2.92; N, 6.43. $C_{16}H_{12}F_4N_2O_3S_2$. Calculated, %: C, 45.71; H, 2.88; N, 6.66. FT-IR (KBr) cm^{-1}: 3346, 3097 (N-H), 3068 (=C-H_{Ar}), 1681 (C=O), 1531 (C=C_{Ar}), 1265 (C=S), 1145 (C-N). LC-MS *m*/*z* (*rel*, %): 420.02 $[M + H]^+$ (100). ^{1}H NMR (400 MHz, DMSO-d_6) δ 12.50 (s, 1H), 12.24 (s, 1H), 8.47 (d, *J* = 6.7 Hz, 1H), 8.23-8.16 (m, 2H), 8.13-8.06 (m, 2H), 7.77 (s, 1H), 7.62 (t, *J* = 9.3 Hz, 1H), 3.32 (s, 3H).

4e:*N*-((4-bromo-2-(trifluoromethyl)phenyl)carbamothioyl)-4-(methylsulfonyl)benzamide

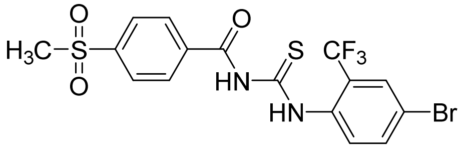

Yield 84%. Light yellow crystal. m.p: 221-223°C. Anal. Found, %: C, 40.11; H, 2.42; N, 5.81. $C_{16}H_{12}BrF_3N_2O_3S_2$. Calculated, %: C, 39.93; H, 2.51; N, 5.82. FT-IR (KBr) cm^{-1}: 3311, 3211 (N-H), 3078 (=C-H_{Ar}), 1677 (C=O), 1550 (C=C_{Ar}), 1257 (C=S), 1151 (C-N). LC-MS *m*/*z* (*rel*, %): 479.94 $[M + H]^+$ (100). ^{1}H NMR (400 MHz, DMSO-d_6) δ 12.42 (s, 1H), 12.29 (s, 1H), 8.25-8.18 (m, 2H), 8.13-8.06 (m, 2H), 8.04-7.95 (m, 2H), 7.76 (d, *J* = 8.5 Hz, 1H), 3.33

(s, 3H).

4f: *N*-((4-fluoro-3-(trifluoromethyl)phenyl)carbamothioyl)-4-(methylsulfonyl)benzamide

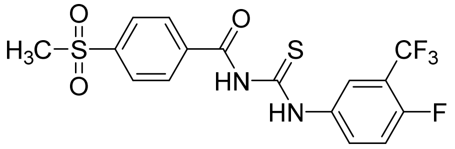

Yield 81%. White crystal. m.p: 205-207°C. Anal. Found, %: C, 45.60; H, 2.79; N, 6.44. $C_{16}H_{12}F_4N_2O_3S_2$. Calculated, %: C, 45.71; H, 2.88; N, 6.66. FT-IR (KBr) cm^{-1}: 3161, 3064 (N-H), 3037 (=C-H_{Ar}), 1697 (C=O), 1544 (C=C_{Ar}), 1216 (C=S), 1141 (C-N). LC-MS *m*/*z* (*rel*, %): 420.02 $[M + H]^+$ (100). ^{1}H NMR (400 MHz, DMSO-d_6) δ 12.37 (s, 1H), 12.05 (s, 1H), 8.21-8.15 (m, 2H), 8.14 (dd, *J* = 6.7, 2.5 Hz, 1H), 8.13-8.06 (m, 2H), 7.96 (dt, *J* = 7.9, 3.6 Hz, 1H), 7.60 (t, *J* = 9.7 Hz, 1H), 3.32 (s, 3H).

4g: 4-(methylsulfonyl)-*N*-((2-(trifluoromethoxy)phenyl)carbamothioyl)benzamide

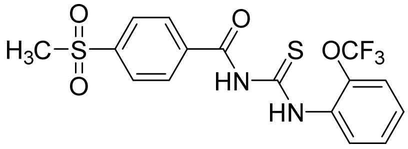

Yield 87%. White solid. m.p: 184-186°C. Anal. Found, %: C, 45.82; H, 3.32; N, 6.61. $C_{16}H_{13}F_3N_2O_4S_2$. Calculated, %: C, 45.93; H, 3.13; N, 6.70. FT-IR (KBr) cm^{-1}: 3228, 3191 (N-H), 3039 (=C-H_{Ar}), 1685 (C=O), 1537 (C=C_{Ar}), 1269 (C=S), 1157 (C-N). LC-MS *m*/*z* (*rel*, %): 418.03 $[M + H]^+$ (100). ^{1}H NMR (400 MHz, DMSO-d_6) δ 12.73 (s, 1H), 12.18 (s, 1H), 8.29 (dd, *J* = 8.0, 1.8 Hz, 1H), 8.23-8.16 (m, 2H), 8.12-8.05 (m, 2H), 7.56-7.40 (m, 3H), 3.32 (s, 3H).

4h: 4-(methylsulfonyl)-*N*-(o-tolylcarbamothioyl)benzamide

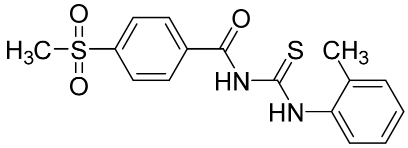

Yield 92%. White crystal. m.p: 190-191°C. Anal. Found, %: C, 55.27; H, 4.51; N, 8.15. $C_{16}H_{16}N_2O_3S_2$. Calculated, %: C, 55.15; H, 4.63; N, 8.04. FT-IR (KBr) cm^{-1}: 3236, 3193 (N-H), 3041 (=C-H_{Ar}), 1689 (C=O), 1546 (C=C_{Ar}), 1265 (C=S), 1149 (C-N). LC-MS *m*/*z* (*rel*, %): 348.06 $[M + H]^+$ (100). ^{1}H NMR (400 MHz, DMSO-d_6) δ 12.16 (s, 1H), 11.94 (s, 1H), 8.24-8.17 (m, 2H), 8.12-8.05 (m, 2H), 7.57 (dd, *J* = 7.3, 2.0 Hz, 1H), 7.32 (dd, *J* = 6.6, 2.4 Hz, 1H), 7.25 (tt, *J* = 7.3, 5.4 Hz, 2H), 3.32 (s, 3H), 2.28 (s, 3H).

4i: N-(4-methylsulfonylbenzoyl)-N'-(3-nitrophenyl) thiourea

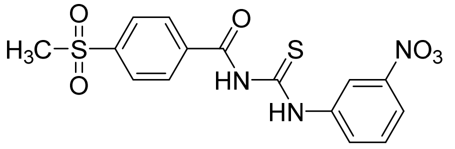

Yield 86%. Light yellow crystal. m.p: 192-193°C. Anal. Found, %: C, 47.60; H, 3.66; N, 11.26. $C_{15}H_{13}N_3O_5S_2$. Calculated, %: C, 47.49; H, 3.45; N, 11.08. FT-IR (KBr) cm^{-1}: 3315, 3259 (N-H), 3004 (=C-H$_{Ar}$), 1670 (C=O), 1542 (C=C$_{Ar}$), 1255 (C=S), 1157 (C-N). LC-MS *m/z* (*rel*, %): 379.03 $[M + H]^+$ (100). ^{1}H NMR (400 MHz, DMSO-*d*$_6$) δ 12.55 (s, 1H), 12.07 (s, 1H), 8.77 (t, *J* = 2.2 Hz, 1H), 8.22-8.02 (m, 6H), 7.73 (t, *J* = 8.1 Hz, 1H), 3.33 (s, 3H).

4j: N-(4-methylsulfonylbenzoyl)-N'-(2,3-dichlorophenyl) thiourea

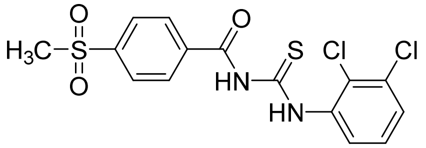

Yield 88%. White solid. m.p: 199-200°C. Anal. Found, %: C, 44.64; H, 3.11; N, 6.87. $C_{15}H_{12}Cl_2N_2O_3S_2$. Calculated, %: C, 44.67; H, 3.00; N, 6.95. FT-IR (KBr) cm^{-1}: 3224, 3188 (N-H), 3088 (=C-H$_{Ar}$), 1685 (C=O), 1533 (C=C$_{Ar}$), 1255 (C=S), 1145 (C-N). LC-MS *m/z* (*rel*, %): 401.97 $[M + H]^+$ (100). ^{1}H NMR (400 MHz, DMSO-*d*$_6$) δ 12.47 (s, 1H), 12.18 (s, 1H), 8.25-8.16 (m, 2H), 8.13-8.05 (m, 2H), 7.88 (dd, *J* = 8.1, 1.5 Hz, 1H), 7.63 (dd, *J* = 8.1, 1.5 Hz, 1H), 7.45 (t, *J* = 8.1 Hz, 1H), 3.32 (s, 3H).

4k: N-(4-methylsulfonylbenzoyl)-N'-(4-methoxyphenyl) thiourea

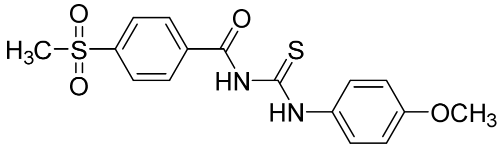

Yield 95%. Light yellow crystal. m.p: 214-215°C. Anal. Found, %: C, 52.65; H, 4.55; N, 7.78. $C_{16}H_{16}N_2O_4S_2$. Calculated, %: C, 52.73; H, 4.43; N, 7.69. FT-IR (KBr) cm^{-1}: 3240, 3191 (N-H), 3010 (=C-H$_{Ar}$), 1672 (C=O), 1533 (C=C$_{Ar}$), 1249 (C=S), 1143 (C-N). LC-MS *m/z* (*rel*, %): 364.06 $[M + H]^+$ (100). ^{1}H NMR (400 MHz, DMSO-*d*$_6$) δ 12.30 (s, 1H), 11.84 (s, 1H), 8.21-8.14 (m, 2H), 8.12-8.04 (m, 2H), 7.61-7.52 (m, 2H), 7.03-6.95 (m, 2H), 3.78 (s, 3H), 3.32 (s, 3H).

4l: N-(4-methylsulfonylbenzoyl)-N'-(2,6-dichlorophenyl) thiourea

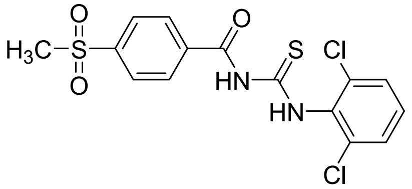

Yield 87%. White crystal. m.p: 201-202°C. Anal. Found, %: C, 44.88; H, 3.12; N, 6.74. $C_{15}H_{12}Cl_2N_2O_3S_2$. Calculated, %: C, 44.67; H, 3.00; N, 6.95. FT-IR (KBr) cm^{-1}: 3201, 3074 (N-H), 3002 (=C-H_{Ar}), 1685 (C=O), 1521 (C=C_{Ar}), 1263 (C=S), 1151 (C-N). LC-MS *m*/*z* (*rel*, %): 401.97 $[M + H]^+$ (100). ^{1}H NMR (400 MHz, DMSO-d_6) δ 12.20 (s, 1H), 11.95 (s, 1H), 8.26-8.19 (m, 2H), 8.12-8.06 (m, 2H), 7.58 (d, *J* = 8.1 Hz, 2H), 7.41 (dd, *J* = 8.6, 7.6 Hz, 1H), 3.33 (s, 3H).

4m: N-(4-methylsulfonylbenzoyl)-N'-(4-nitrophenyl) thiourea

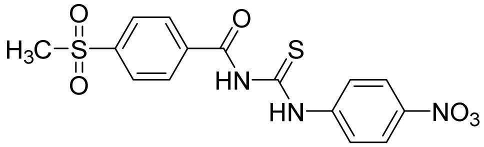

Yield 89%. Yellow crystal. m.p: 224-225°C. Anal. Found, %: C, 47.68; H, 3.37; N, 11.29. $C_{15}H_{13}N_3O_5S_2$. Calculated, %: C, 47.49; H, 3.45; N, 11.08. FT-IR (KBr) cm^{-1}: 3225, 3022 (N-H), 3008 (=C-H_{Ar}), 1674 (C=O), 1535 (C=C_{Ar}), 1269 (C=S), 1145 (C-N). LC-MS *m*/*z* (*rel*, %): 379.03 $[M + H]^+$ (100). ^{1}H NMR (400 MHz, DMSO-d_6) δ 12.67 (s, 1H), 12.07 (s, 1H), 8.35-8.26 (m, 2H), 8.22-8.15 (m, 2H), 8.13-8.02 (m, 4H), 3.32 (s, 3H).

4n: N-(4-methylsulfonylbenzoyl)-N'-(2,5-dichlorophenyl) thiourea

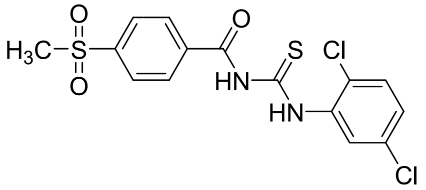

Yield 87%. White solid. m.p: 184-185°C. Anal. Found, %: C, 44.46; H, 3.22; N, 6.91. $C_{15}H_{12}Cl_2N_2O_3S_2$. Calculated, %: C, 44.67; H, 3.00; N, 6.95. FT-IR (KBr) cm^{-1}: 3359, 3267 (N-H), 3006 (=C-H_{Ar}), 1681 (C=O), 1541 (C=C_{Ar}), 1251 (C=S), 1143 (C-N). LC-MS *m*/*z* (*rel*, %): 401.97 $[M + H]^+$ (100). ^{1}H NMR (400 MHz, DMSO-d_6) δ 12.60 (s, 1H), 12.23 (s, 1H), 8.20 (dd, *J* = 8.1, 2.1 Hz, 3H), 8.13-8.06 (m, 2H), 7.65 (d, *J* = 8.6 Hz, 1H), 7.44 (dd, *J* = 8.7, 2.6 Hz, 1H), 3.32 (s, 3H).

4o: N-(4-methylsulfonylbenzoyl)-N'-(3-chlorophenyl) thiourea

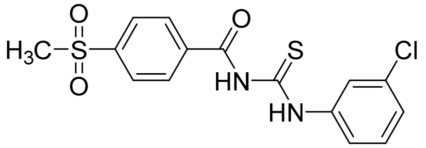

Yield 85%. Light yellow crystal. m.p: 188-189°C. Anal. Found, %: C, 48.64; H, 3.37; N, 7.78. $C_{15}H_{13}ClN_2O_3S_2$. Calculated, %: C, 48.85; H, 3.55; N, 7.59. FT-IR (KBr) cm^{-1}: 3359, 3064 (N-H), 3008 (=C-H_{Ar}), 1674 (C=O), 1533 (C=C_{Ar}), 1257 (C=S), 1145 (C-N). LC-MS *m/z* (*rel*, %): 368.01 $[M + H]^+$ (100). ^1H NMR (400 MHz, DMSO-d_6) δ 12.43 (s, 1H), 11.97 (s, 1H), 8.17 (d, *J* = 8.5 Hz, 2H), 8.09 (d, *J* = 8.5 Hz, 2H), 7.93 (s, 1H), 7.58 (d, *J* = 9.0 Hz, 1H), 7.46 (t, *J* = 8.0 Hz, 1H), 7.36 (dt, *J* = 8.0, 1.6 Hz, 1H), 3.32 (s, 3H).

4p: N-(4-methylsulfonylbenzoyl)-N'-(2,4,5-trifluorophenyl) thiourea

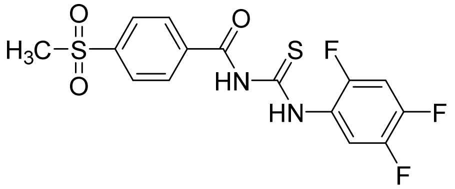

Yield 87%. White crystal. m.p: 211-212°C. Anal. Found, %: C, 46.08; H, 2.94; N, 7.01. $C_{15}H_{11}F_3N_2O_3S_2$. Calculated, %: C, 46.39; H, 2.85; N, 7.21. FT-IR (KBr) cm^{-1}: 3390, 3087 (N-H), 3008 (=C-H_{Ar}), 1677 (C=O), 1546 (C=C_{Ar}), 1205 (C=S), 1155 (C-N). LC-MS *m/z* (*rel*, %): 388.02 $[M + H]^+$ (100). ^1H NMR (400 MHz, DMSO-d_6) δ 1H NMR (400 MHz, DMSO-d6) δ 12.48 (s, 1H), 12.02 (s, 1H), 8.20 (d, *J* = 8.5 Hz, 2H), 8.12 (d, *J* = 8.5 Hz, 2H), 7.84 (ddd, *J* = 11.6, 8.6, 7.0 Hz, 1H), 7.73 (td, *J* = 10.5, 7.4 Hz, 1H), 3.31 (s, 3H).

4q: N-(4-methylsulfonylbenzoyl)-N'-(4-chlorophenyl) thiourea

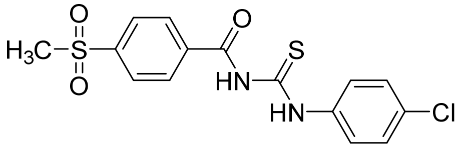

Yield 90%. Light yellow crystal. m.p: 212-213°C. Anal. Found, %: C, 48.91; H, 3.67; N, 7.28. $C_{15}H_{13}ClN_2O_3S_2$. Calculated, %: C, 48.85; H, 3.55; N, 7.59. FT-IR (KBr) cm^{-1}: 3238, 3039 (N-H), 3014 (=C-H_{Ar}), 1672 (C=O), 1529 (C=C_{Ar}), 1261 (C=S), 1143 (C-N). LC-MS *m/z* (*rel*, %): 368.01 $[M + H]^+$ (100). ^1H NMR (400 MHz, DMSO-d_6) δ 12.40 (s, 1H), 11.94 (s, 1H), 8.17 (d, *J* = 8.5 Hz, 2H), 8.12-8.05 (m, 2H), 7.72 (d, *J* = 8.4 Hz, 2H), 7.54-7.45 (m, 2H), 3.32 (s, 3H).

4r: N-(4-methylsulfonylbenzoyl)-N'-(2,4-dichlorophenyl) thiourea

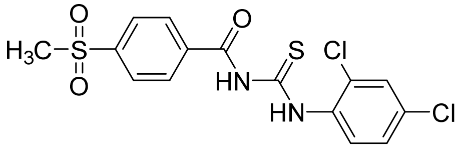

Yield 83%. White crystal. m.p: 194-195°C. Anal. Found, %: C, 44.55; H, 3.21; N, 6.65. $C_{15}H_{12}Cl_2N_2O_3S_2$. Calculated, %: C, 44.67; H, 3.00; N, 6.95. FT-IR (KBr) cm^{-1}: 3382, 3097 (N-H), 3006 (=C-H_{Ar}), 1683 (C=O), 1537 (C=C_{Ar}), 1251 (C=S), 1145 (C-N). LC-MS *m/z* (*rel*, %): 401.97 $[M + H]^+$ (100). ^{1}H NMR (400 MHz, DMSO-d_6) δ 12.46 (s, 1H), 12.18 (s, 1H), 8.23-8.16 (m, 2H), 8.12-8.05 (m, 2H), 8.00 (d, *J* = 8.7 Hz, 1H), 7.80 (d, *J* = 2.4 Hz, 1H), 7.52 (dd, *J* = 8.7, 2.4 Hz, 1H), 3.32 (s, 3H).

4s: N-(4-methylsulfonylbenzoyl)-N'-(4-methylphenyl) thiourea

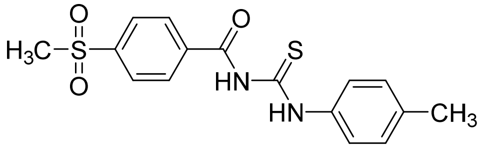

Yield 84%.Light yellow crystal. m.p: 268-269°C. Anal. Found, %: C, 55.34; H, 4.85; N, 8.24. $C_{16}H_{16}N_2O_3S_2$. Calculated, %: C, 55.15; H, 4.63; N, 8.04. FT-IR (KBr) cm^{-1}: 3245, 3037 (N-H), 3012 (=C-H_{Ar}), 1674 (C=O), 1525 (C=C_{Ar}), 1263 (C=S), 1143 (C-N). LC-MS *m/z* (*rel*, %): 348.06 $[M + H]^+$ (100). ^{1}H NMR (400 MHz, DMSO-d_6) δ 12.38 (s, 1H), 11.85 (s, 1H), 8.20-8.14 (m, 2H), 8.12-8.05 (m, 2H), 7.56 (d, *J* = 8.2 Hz, 2H), 7.24 (d, *J* = 8.1 Hz, 2H), 3.31 (s, 3H), 2.33 (s, 3H).

4t: N-(4-methylsulfonylbenzoyl)-N'-(3-methylphenyl) thiourea

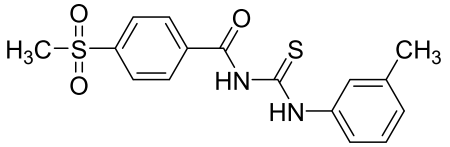

Yield 88%. Light yellow crystal. m.p: 172.5-173.5°C. Anal. Found, %: C, 55.24; H, 4.73; N, 8.25. $C_{16}H_{16}N_2O_3S_2$. Calculated, %: C, 55.15; H, 4.63; N, 8.04. FT-IR (KBr) cm^{-1}: 3348, 3064 (N-H), 3006 (=C-H_{Ar}), 1674 (C=O), 1525 (C=C_{Ar}), 1263 (C=S), 1143 (C-N). LC-MS *m/z* (*rel*, %): 348.06 $[M + H]^+$ (100). ^{1}H NMR (400 MHz, DMSO-d_6) δ 12.43 (s, 1H), 11.87 (s, 1H), 8.17 (d, *J* = 8.3 Hz, 2H), 8.11-8.05 (m, 2H), 7.56-7.47 (m, 2H), 7.32 (t, *J* = 7.8 Hz, 1H), 7.11 (d, *J* = 7.6 Hz, 1H), 3.32 (s, 3H), 2.34 (s, 3H).

4u: N-(4-methylsulfonylbenzoyl)-N'-(2-nitrophenyl) thiourea

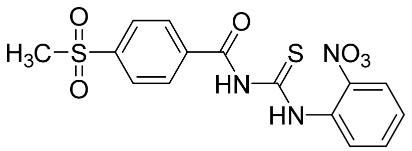

Yield 91%. Yellow solid. m.p: 192-193°C. Anal. Found, %: C, 47.68; H, 3.37; N, 11.27. $C_{15}H_{13}N_3O_5S_2$. Calculated, %: C, 47.49; H, 3.45; N, 11.08. FT-IR (KBr) cm^{-1}: 3292, 3018 (N-H), 3006 (=C-H_{Ar}), 1672 (C=O), 1519 (C=C_{Ar}), 1257 (C=S), 1153 (C-N). LC-MS *m/z* (*rel*, %): 379.03 $[M + H]^+$ (100). ^{1}H NMR (400 MHz, DMSO-d_6) δ 12.72 (s, 1H), 12.19 (s, 1H), 8.24-8.17 (m, 2H), 8.13 (dd, J = 8.2, 1.5 Hz, 1H), 8.13-8.06 (m, 2H), 7.96 (dd, J = 8.2, 1.4 Hz, 1H), 7.81 (td, J = 7.7, 1.5 Hz, 1H), 7.58 (td, J = 7.9, 7.5, 1.4 Hz, 1H), 3.32 (s, 3H).

4v: N-(4-methylsulfonylbenzoyl)-N'-(phenyl) thiourea

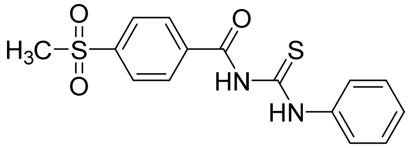

Yield 89%. Light yellow crystal. m.p: 187-188°C. Anal. Found, %: C, 53.69; H, 4.41; N, 8.71. $C_{15}H_{14}N_2O_3S_2$. Calculated, %: C, 53.88; H, 4.22; N, 8.38. FT-IR (KBr) cm^{-1}: 3249, 3037 (N-H), 3014 (=C-H_{Ar}), 1670 (C=O), 1525 (C=C_{Ar}), 1265 (C=S), 1141 (C-N). LC-MS *m/z* (*rel*, %): 334.04 $[M + H]^+$ (100). ^{1}H NMR (400 MHz, DMSO-d_6) δ 12.44 (s, 1H), 11.89 (s, 1H), 8.18 (d, J = 8.3 Hz, 2H), 8.12-8.05 (m, 2H), 7.69 (d, J = 7.9 Hz, 2H), 7.44 (t, J = 7.8 Hz, 2H), 7.29 (t, J = 7.4 Hz, 1H), 3.32 (s, 3H).

4w: N-(4-methylsulfonylbenzoyl)-N'-(2,4-dimethylphenyl) thiourea

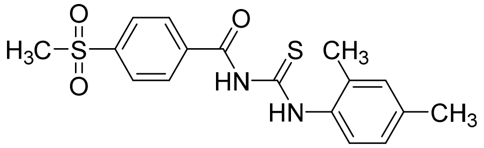

Yield 87%. White crystal. m.p: 221-223°C. Anal. Found, %: C, 56.51; H, 5.33; N, 7.52. $C_{17}H_{18}N_2O_3S_2$. Calculated, %: C, 56.33; H, 5.01; N, 7.73. FT-IR (KBr) cm^{-1}: 3217, 3041 (N-H), 3002 (=C-H_{Ar}), 1674 (C=O), 1531 (C=C_{Ar}), 1265 (C=S), 1147 (C-N). LC-MS *m/z* (*rel*, %): 362.08 $[M + H]^+$ (100). ^{1}H NMR (400 MHz, DMSO-d_6) δ 12.08 (s, 1H), 11.90 (s, 1H), 8.22-8.16 (m, 2H), 8.12-8.05 (m, 2H), 7.43 (d, J = 8.0 Hz, 1H), 7.13 (d, J = 2.1 Hz, 1H), 7.06 (dd, J = 8.0, 2.1 Hz, 1H), 3.32 (s, 3H), 2.30 (s, 3H), 2.23 (s, 3H).

2. IR and 1H NMR spectra of acylthiourea derivatives (4a - 4w)

4a：N-(4-methylsulfonylbenzoyl)-N’-(2-fluoro-4-chlorophenyl) thiourea

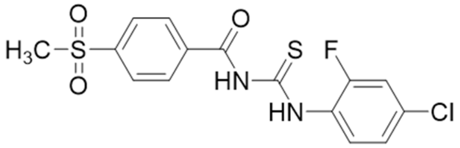

IR

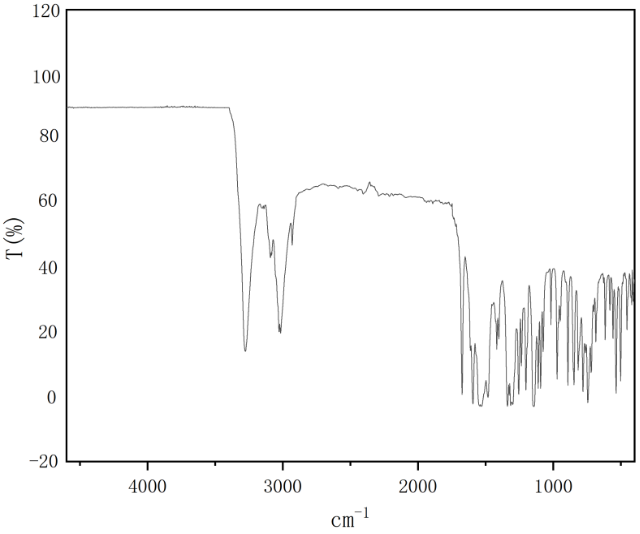

^{1}H NMR

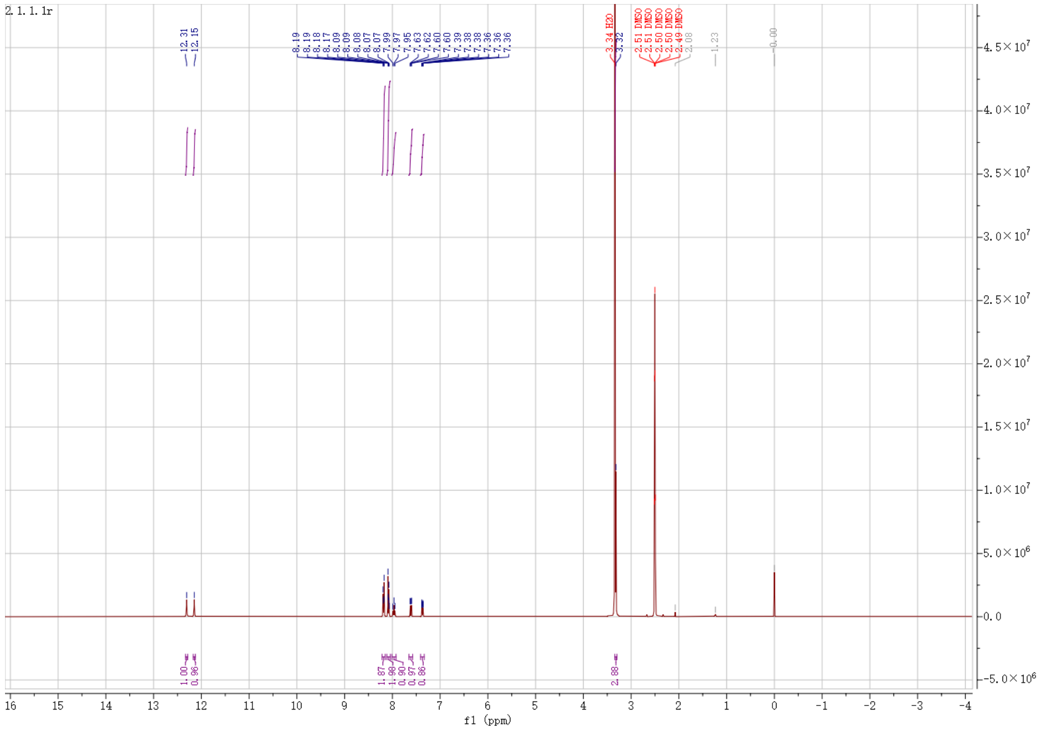

4b：N-(4-methylsulfonylbenzoyl)-N’-(3-trifluoromethyl-4-chlorophenyl) thiourea

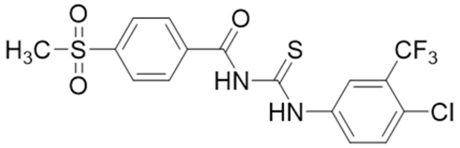

IR

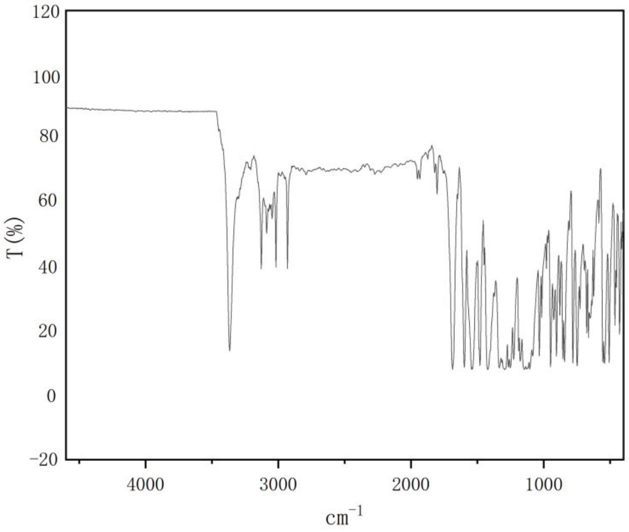

^{1}H NMR

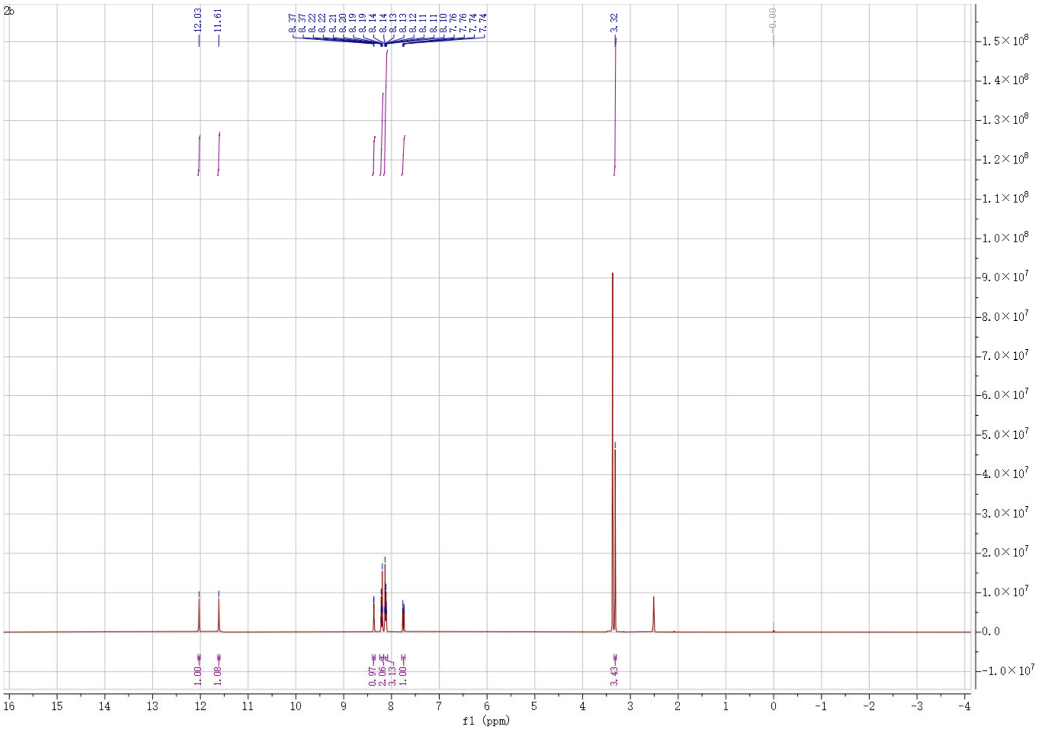

4c：N-(4-methylsulfonylbenzoyl)-N'-(4-iodophenyl) thiourea

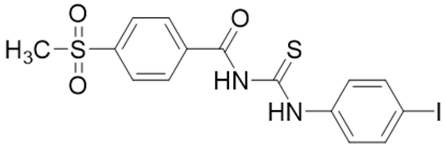

IR

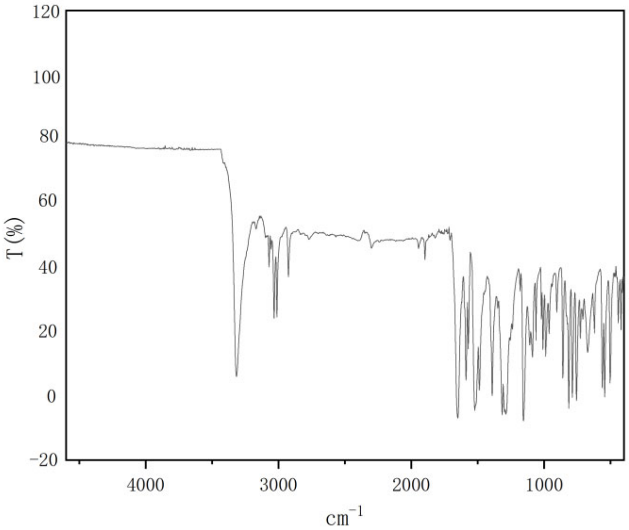

^{1}H NMR

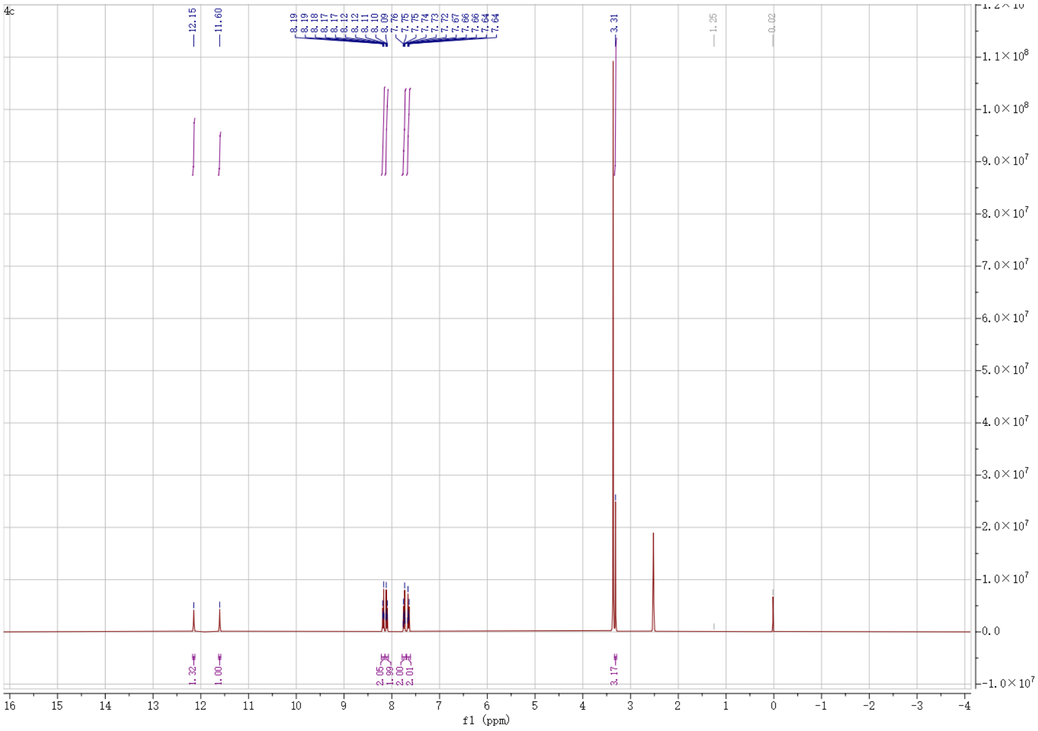

4d：N-(4-methylsulfonylbenzoyl)-N’-(2-fluoro-5-trifluoromethylphenyl) thiourea

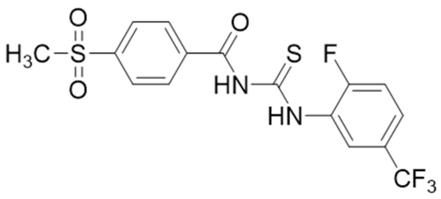

IR

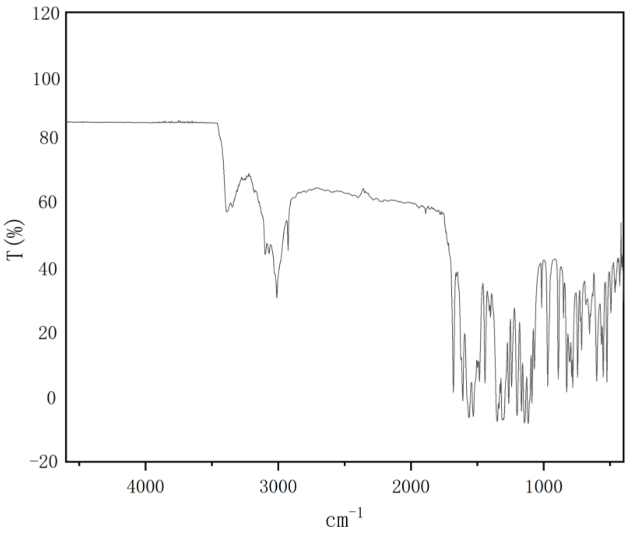

^{1}H NMR

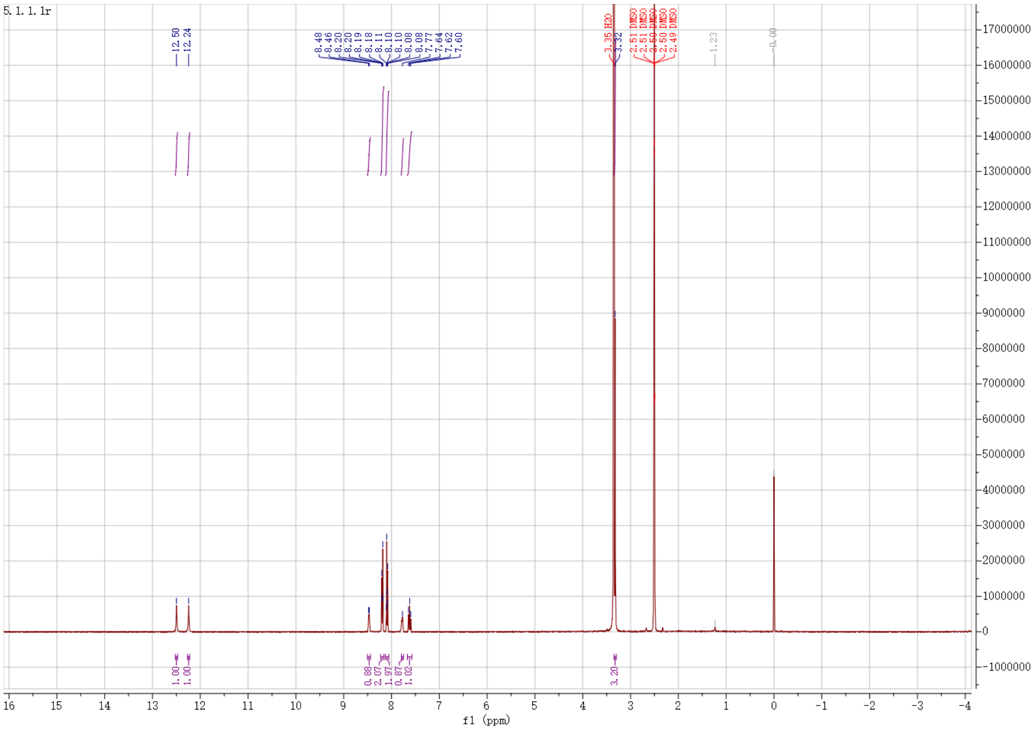

4e：N-(4-methylsulfonylbenzoyl)-N'-(2-trifluoromethyl-4-bromophenyl) thiourea

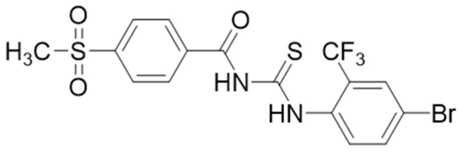

IR

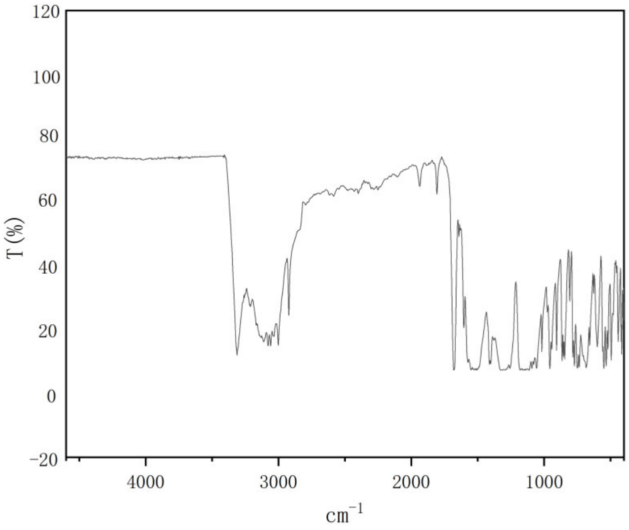

^{1}H NMR

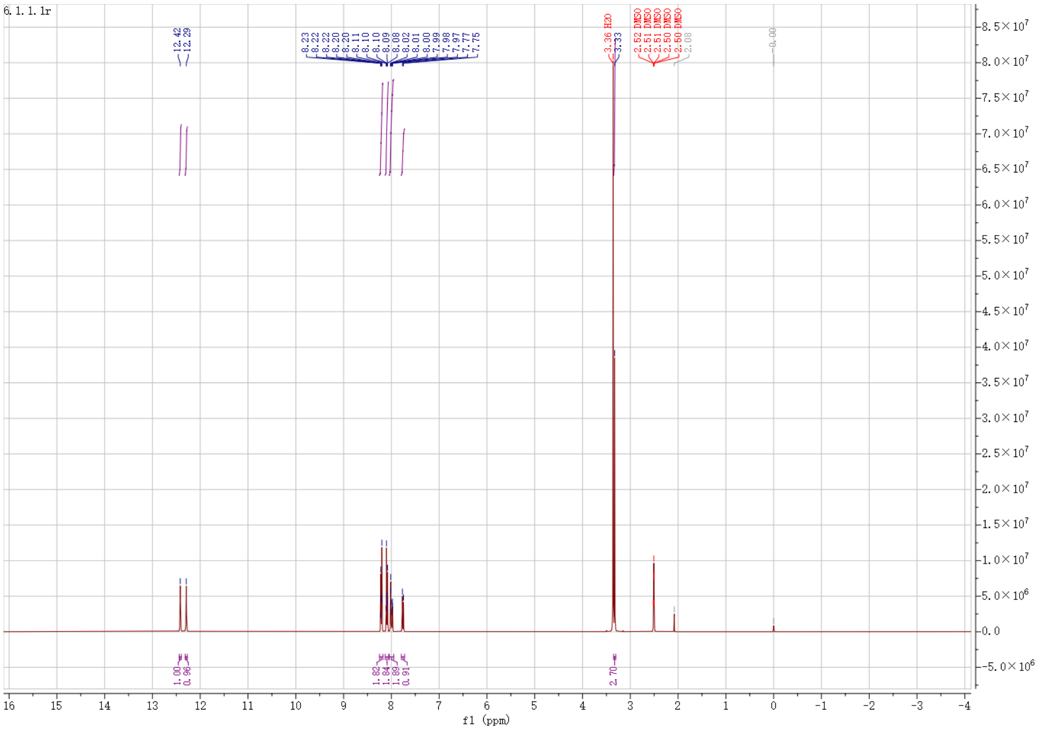

4f：N-(4-methylsulfonylbenzoyl)-N'-(3-trifluoromethyl-4-fluorophenyl) thiourea

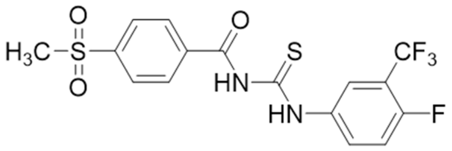

IR

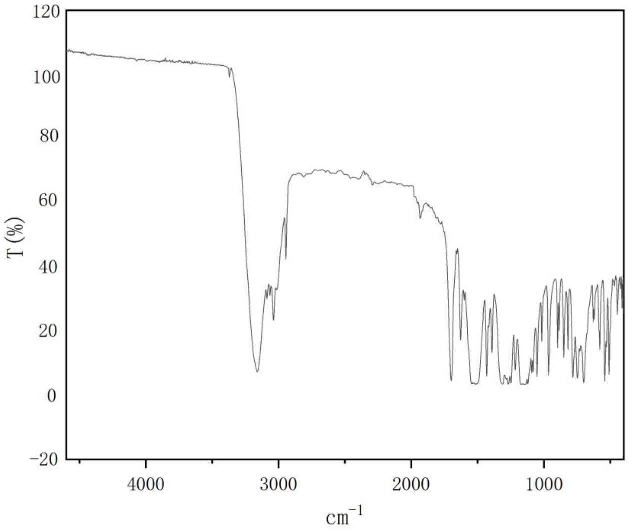

^{1}H NMR

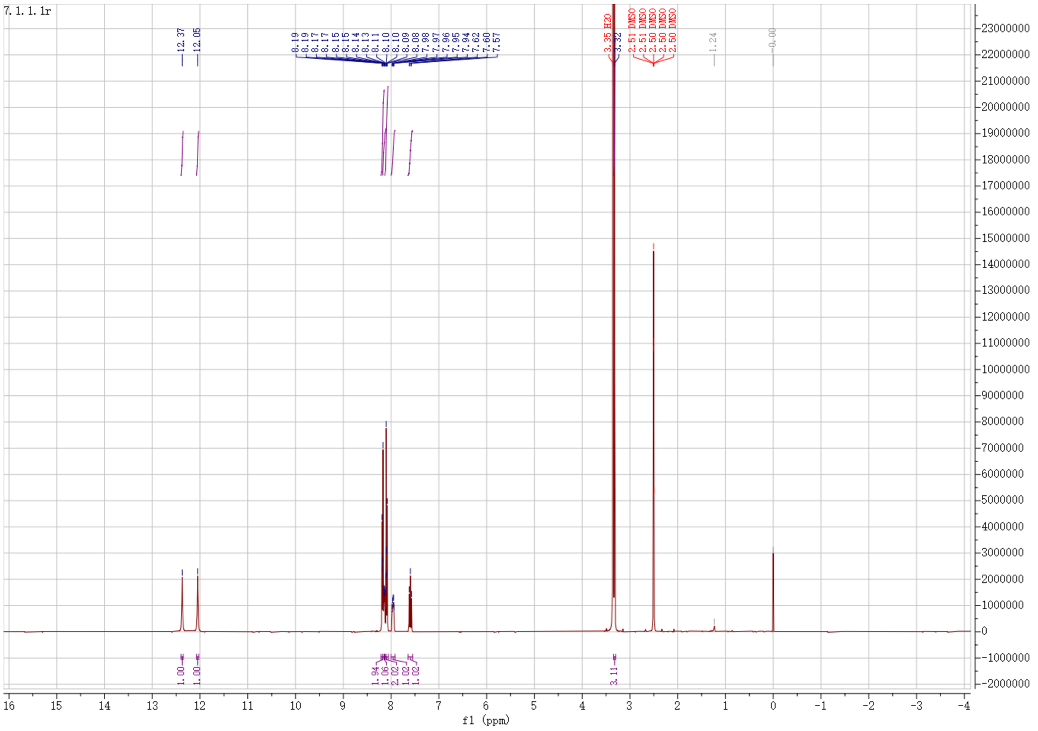

4g：N-(4-methylsulfonylbenzoyl)-N’-(2-trifluoromethoxyphenyl) thiourea

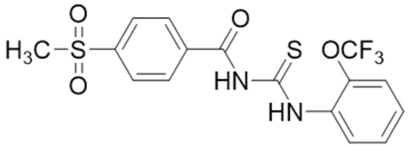

IR

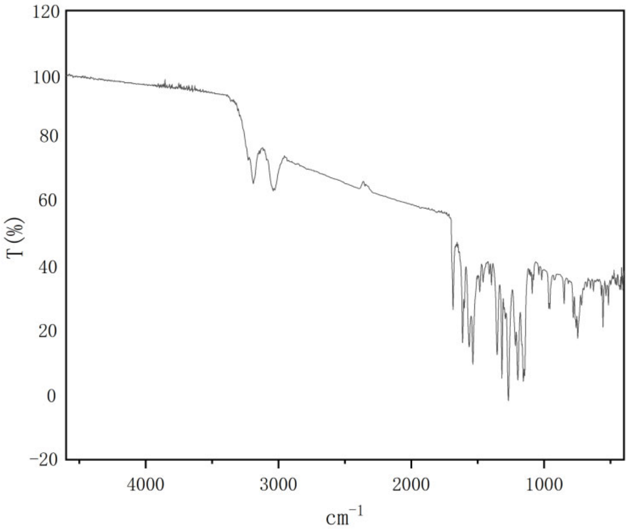

^{1}H NMR

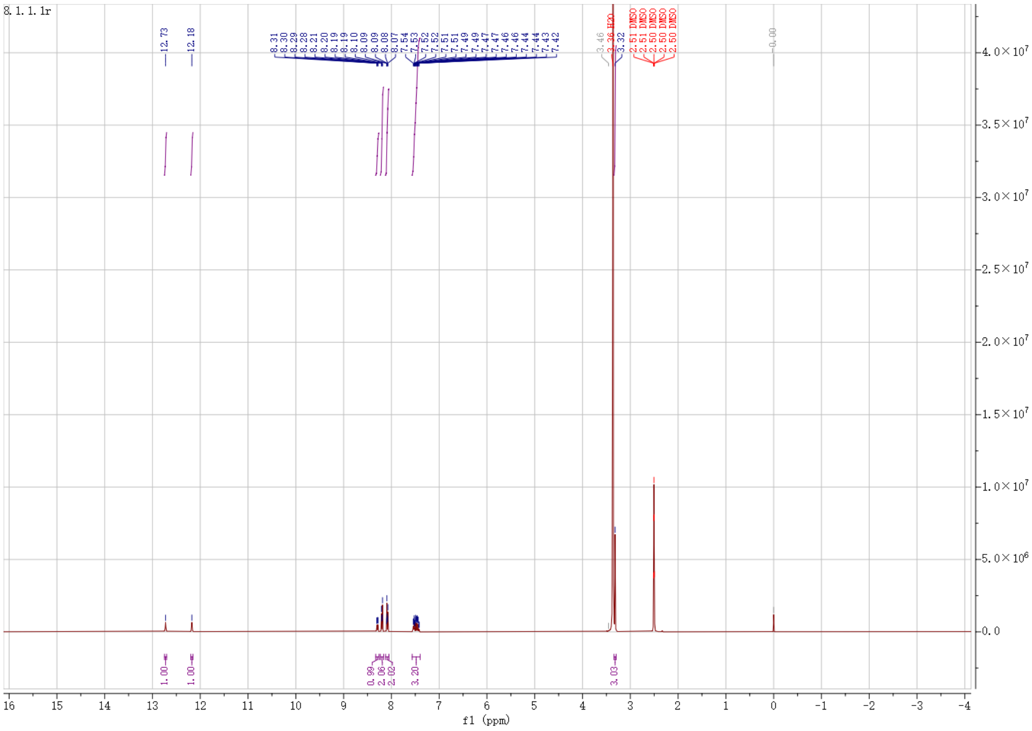

4h：N-(4-methylsulfonylbenzoyl)-N’-(2-methylphenyl) thiourea

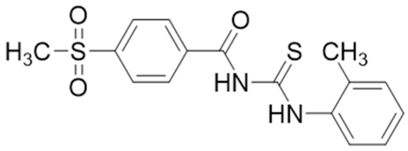

IR

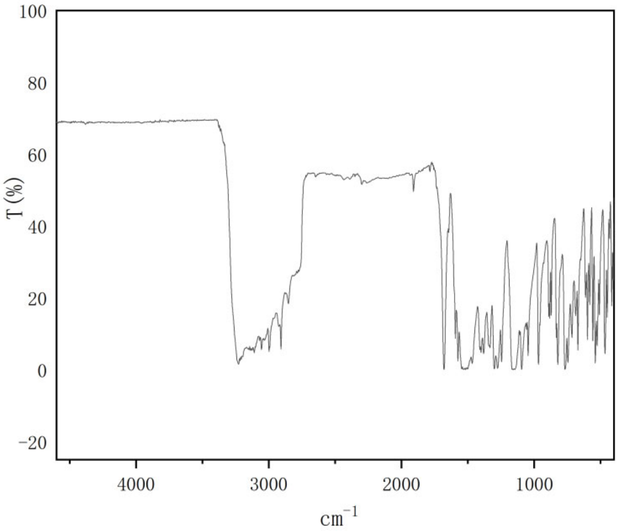

^{1}H NMR

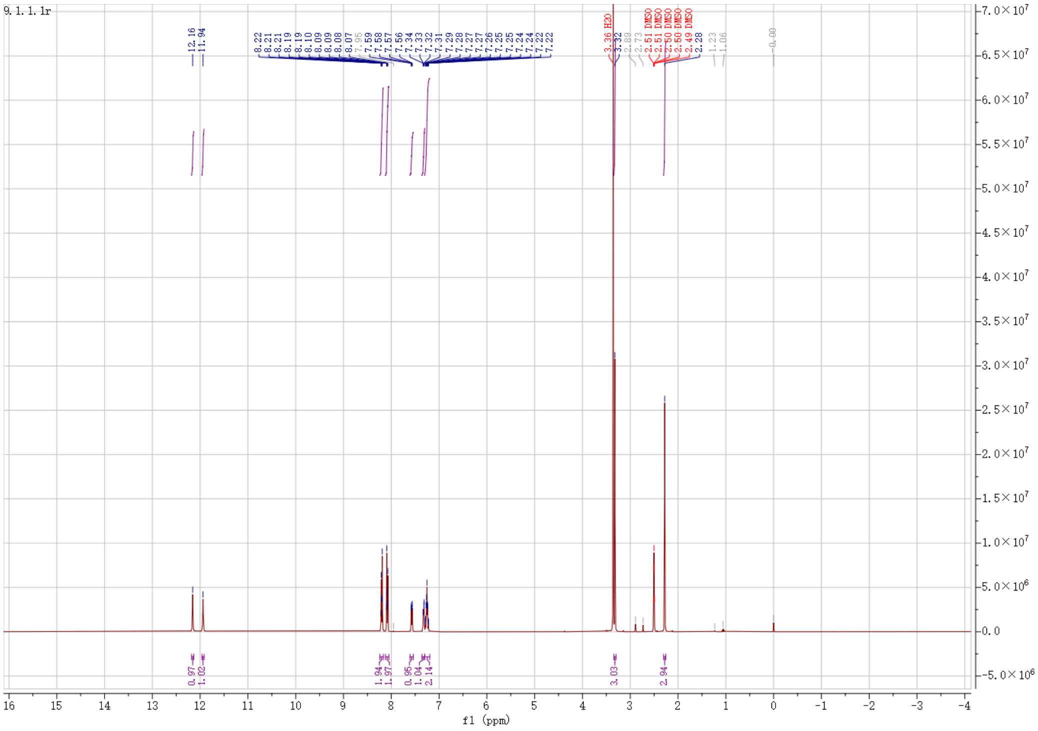

4i: N-(4-methylsulfonylbenzoyl)-N'-(3-nitrophenyl) thiourea

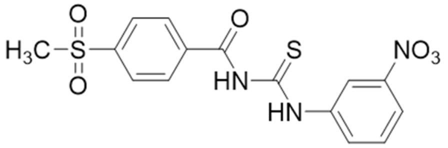

IR

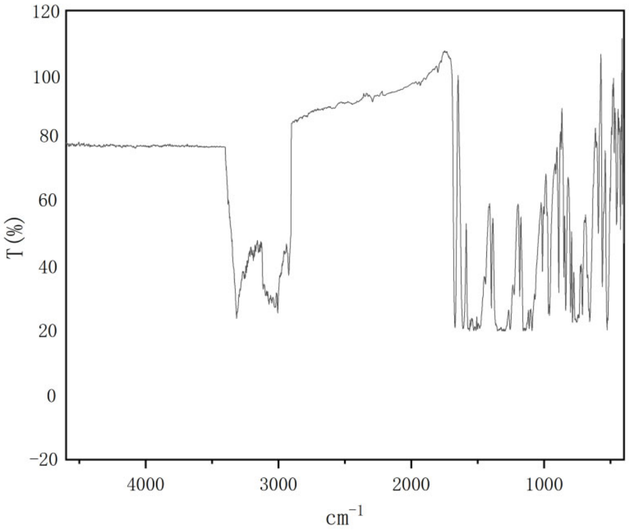

^{1}H NMR

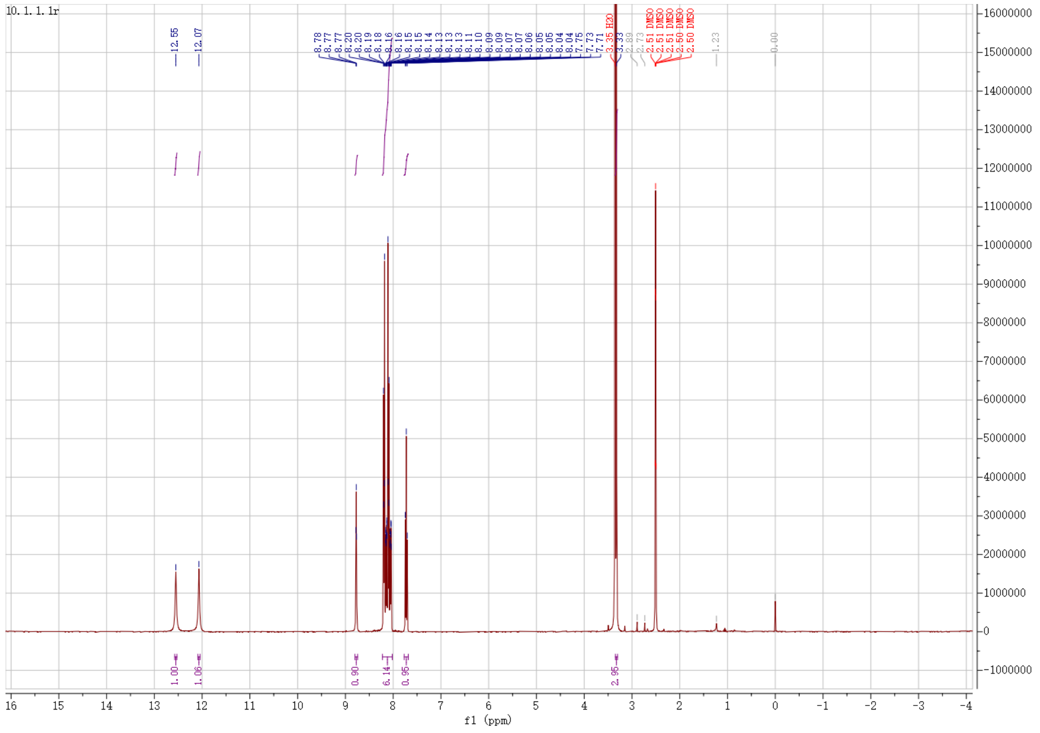

4j：N-(4-methylsulfonylbenzoyl)-N’-(2,3-dichlorophenyl) thiourea

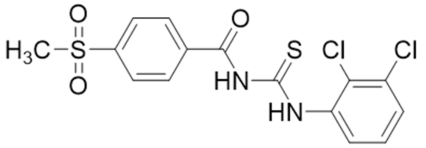

IR

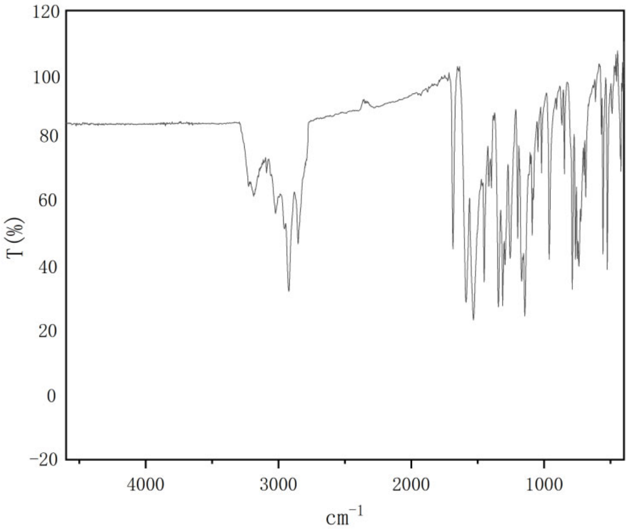

^{1}H NMR

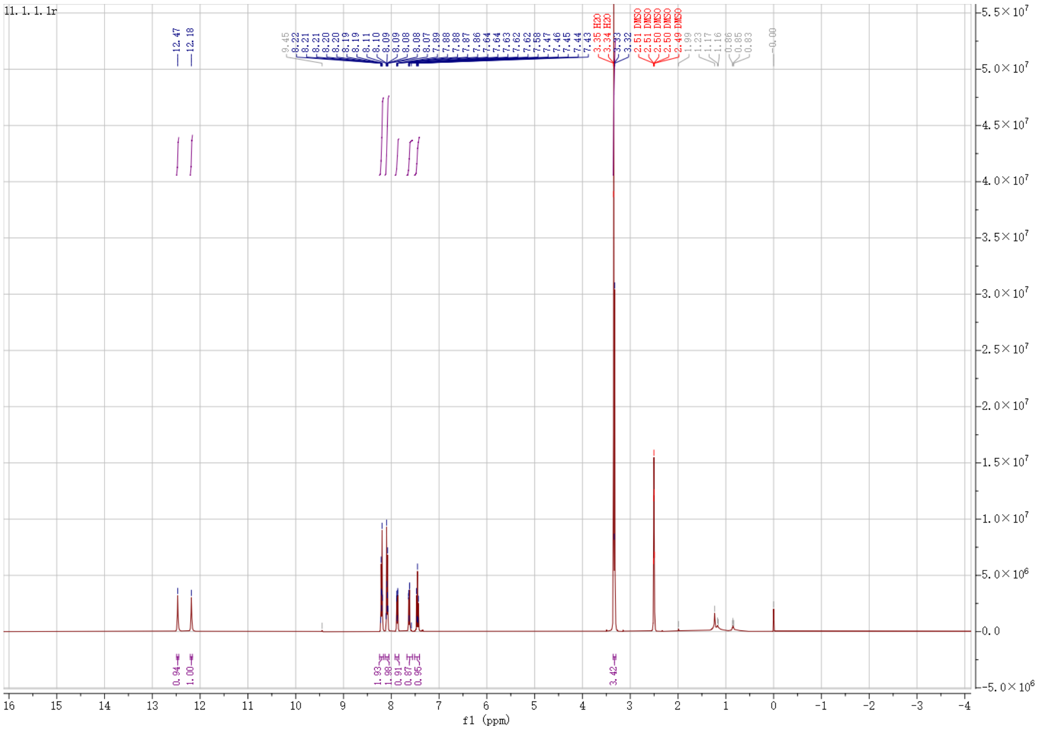

4k：N-(4-methylsulfonylbenzoyl)-N’-(4-methoxyphenyl) thiourea

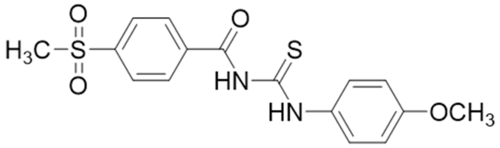

IR

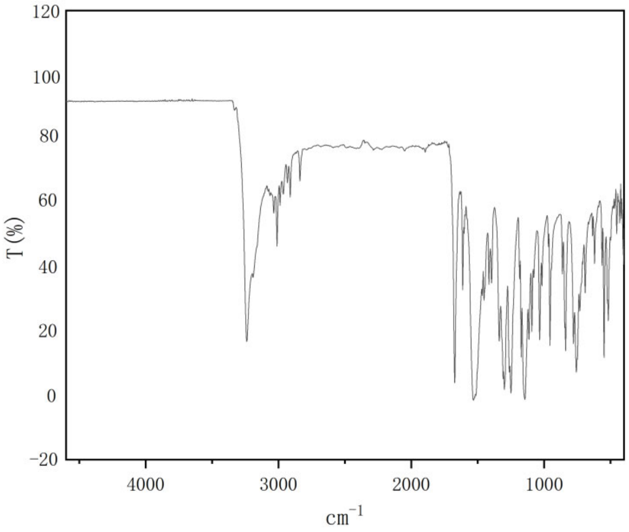

^{1}H NMR

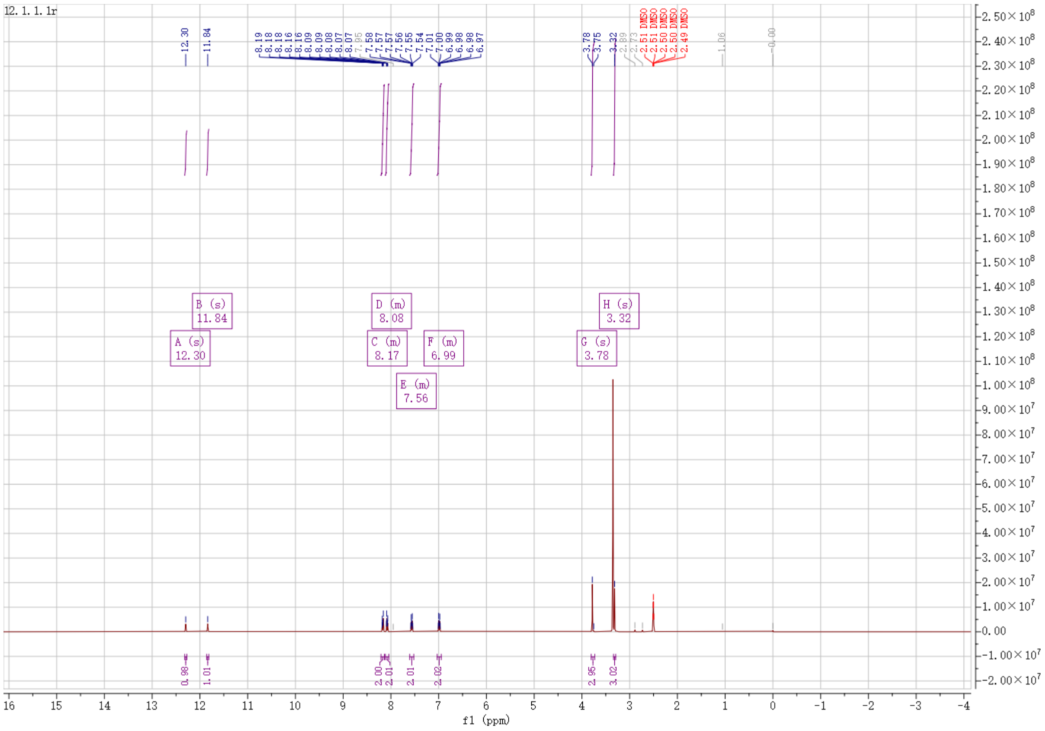

4l: N-(4-methylsulfonylbenzoyl)-N'-(2,6-dichlorophenyl) thiourea

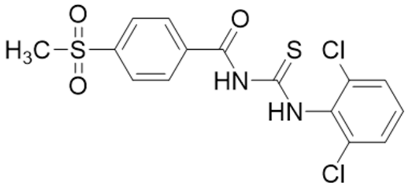

IR

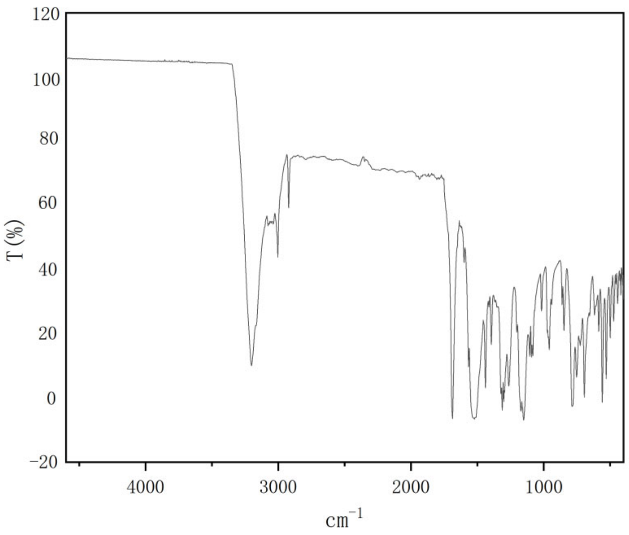

^{1}H NMR

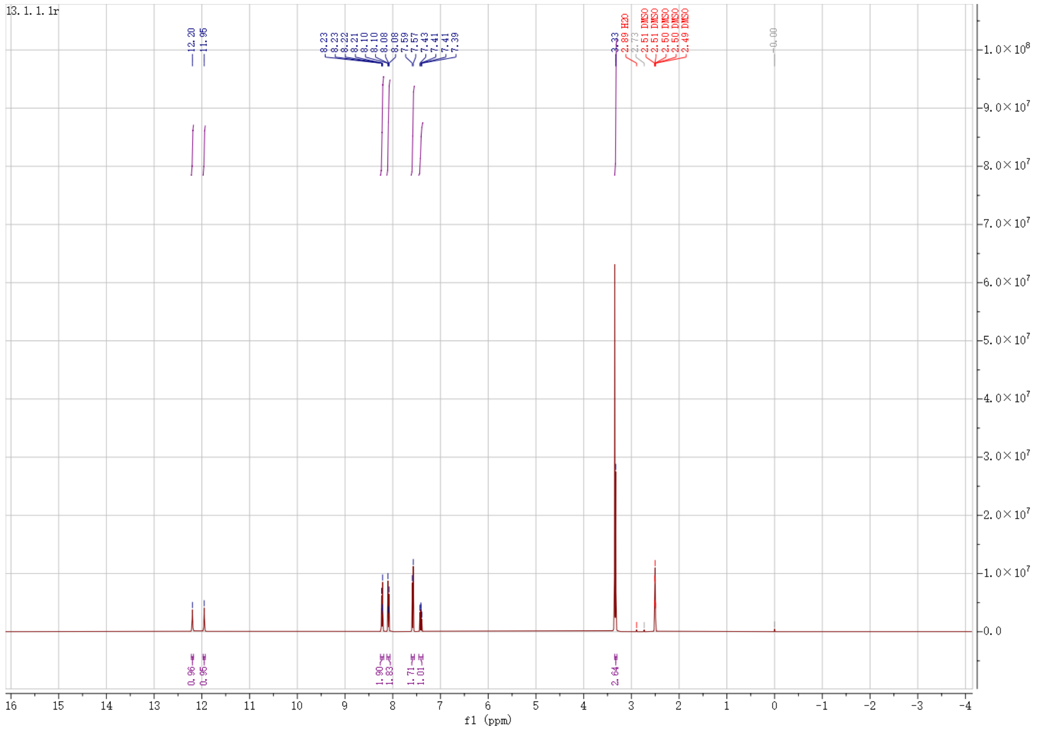

4m：N-(4-methylsulfonylbenzoyl)-N'-(4-nitrophenyl) thiourea

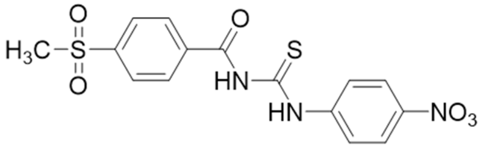

IR

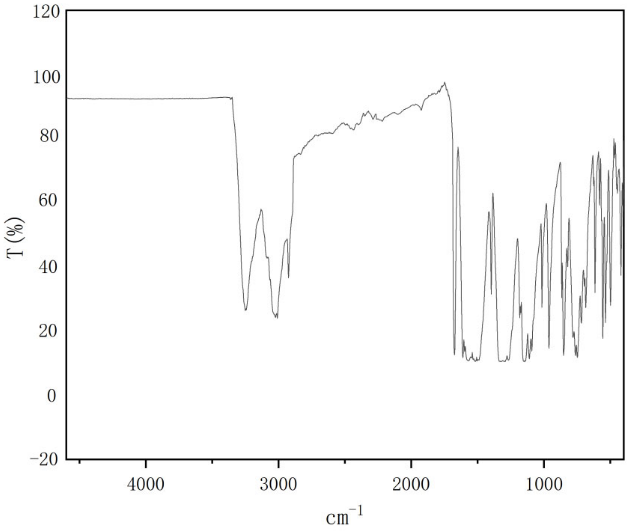

^{1}H NMR

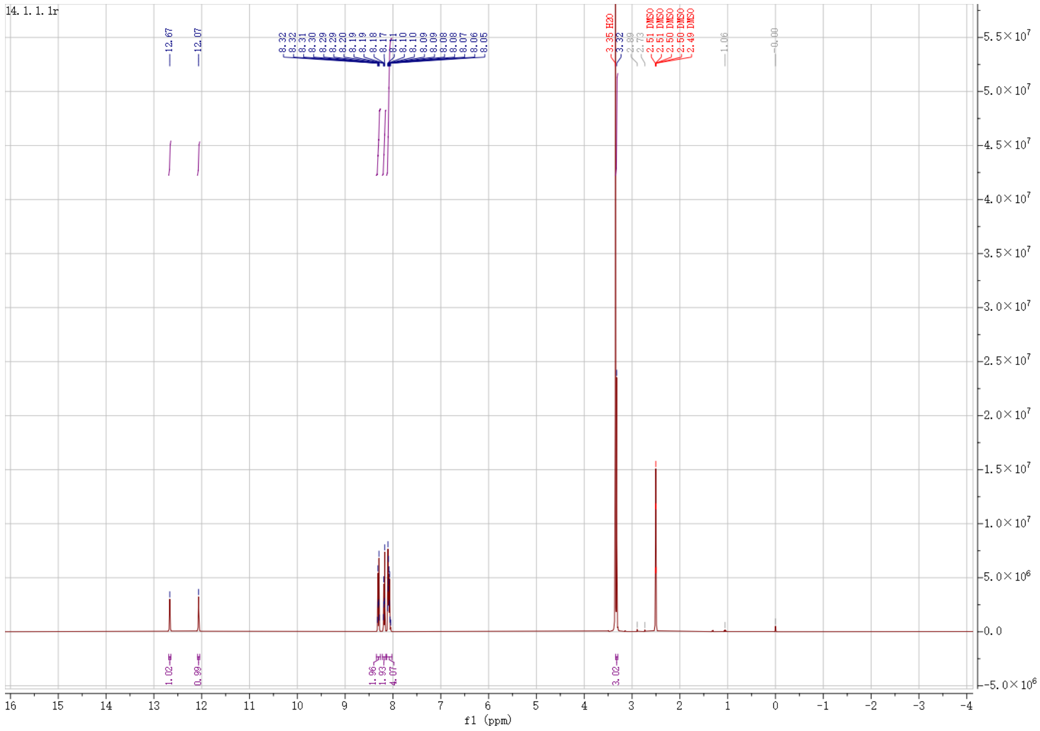

4n：N-(4-methylsulfonylbenzoyl)-N'-(2,5-dichlorophenyl) thiourea

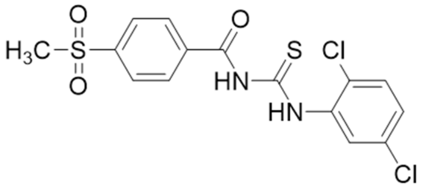

IR

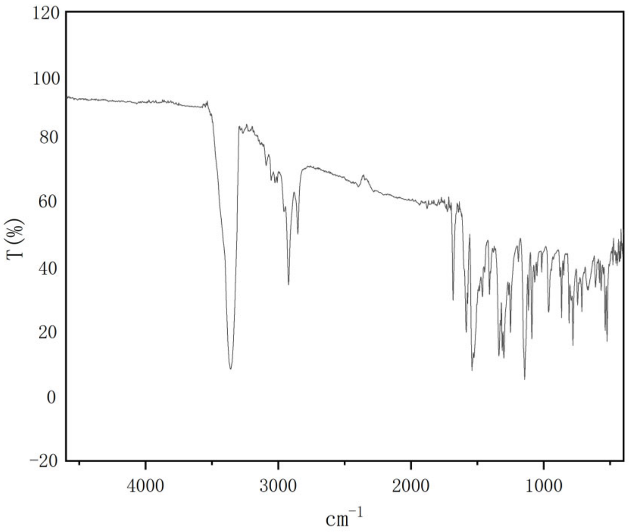

^{1}H NMR

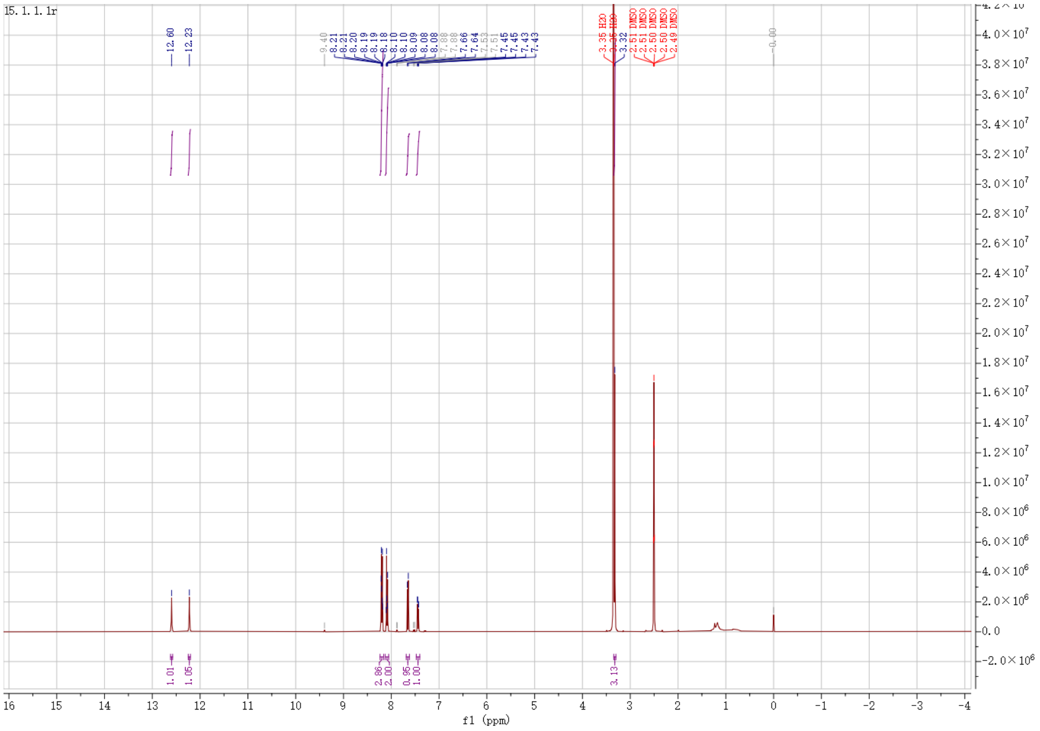

4o：N-(4-methylsulfonylbenzoyl)-N’-(3-chlorophenyl) thiourea

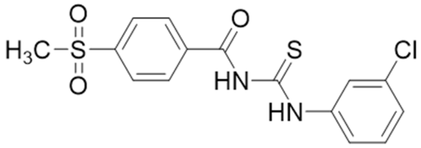

IR

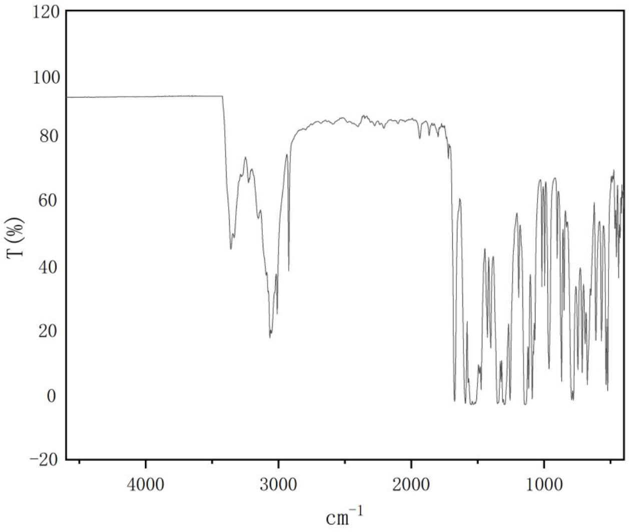

^{1}H NMR

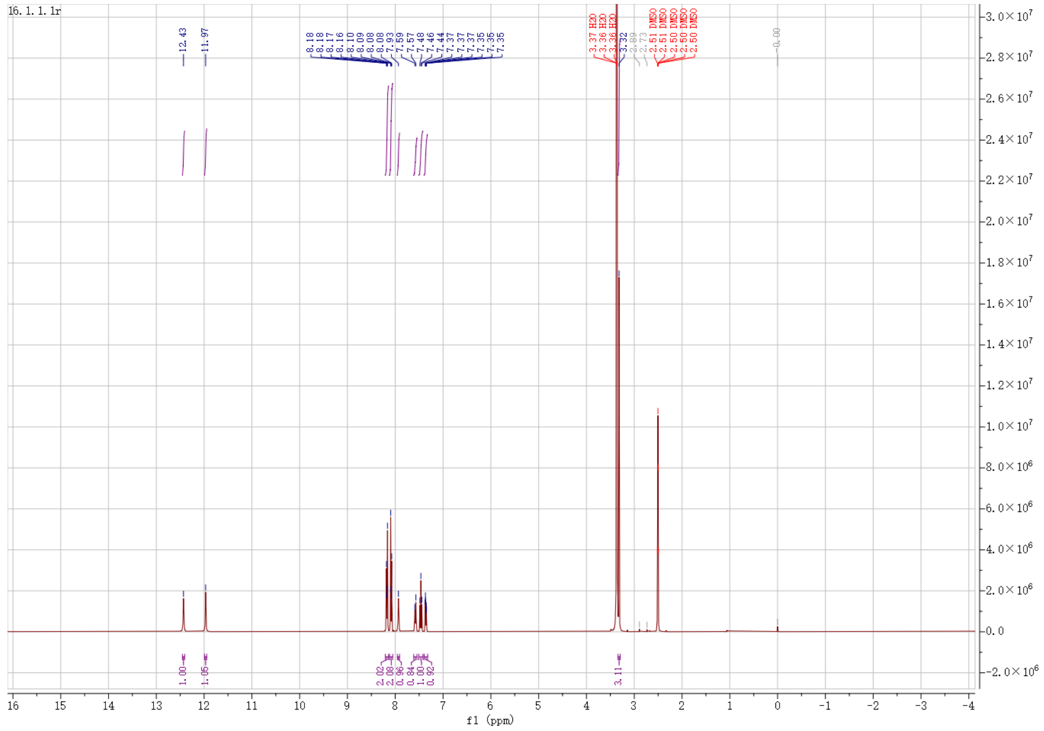

4p：N-(4-methylsulfonylbenzoyl)-N'-(2,4,5-trifluorophenyl) thiourea

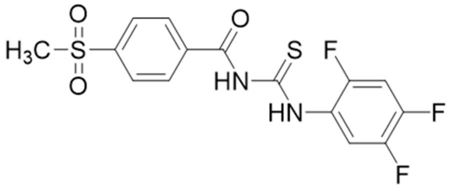

IR

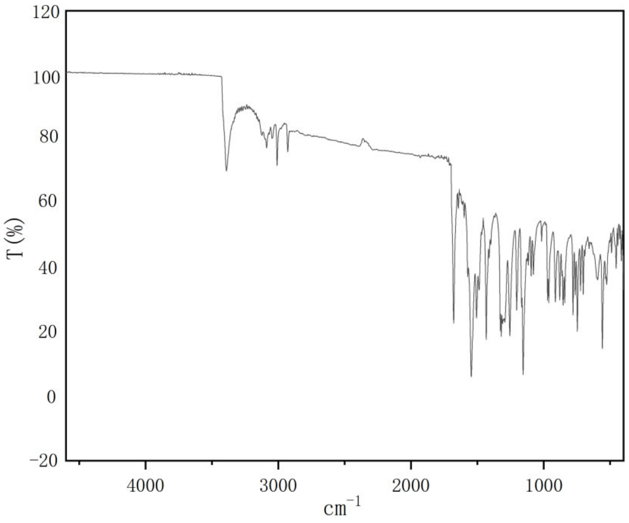

^{1}H NMR

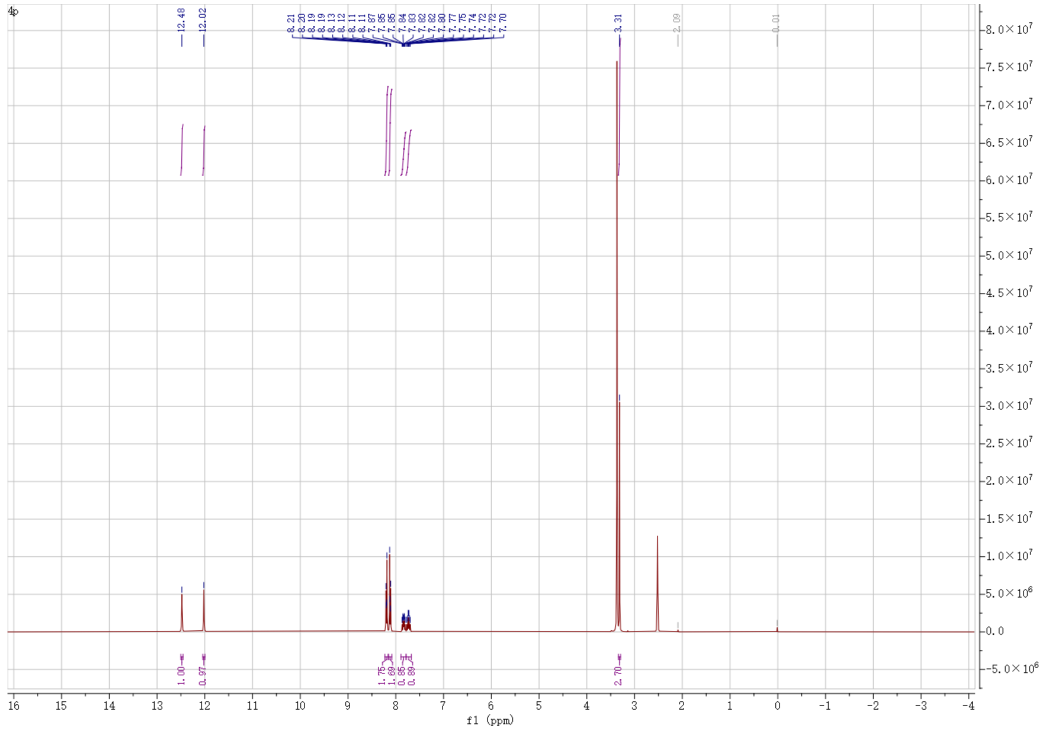

4q：N-(4-methylsulfonylbenzoyl)-N'-(4-chlorophenyl) thiourea

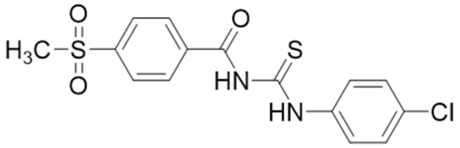

IR

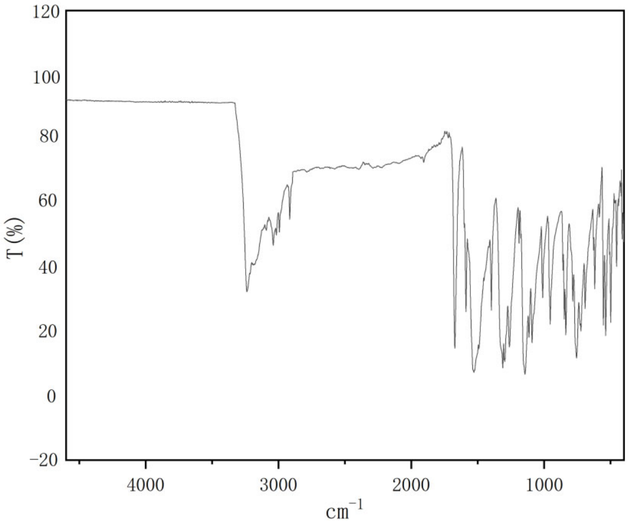

^{1}H NMR

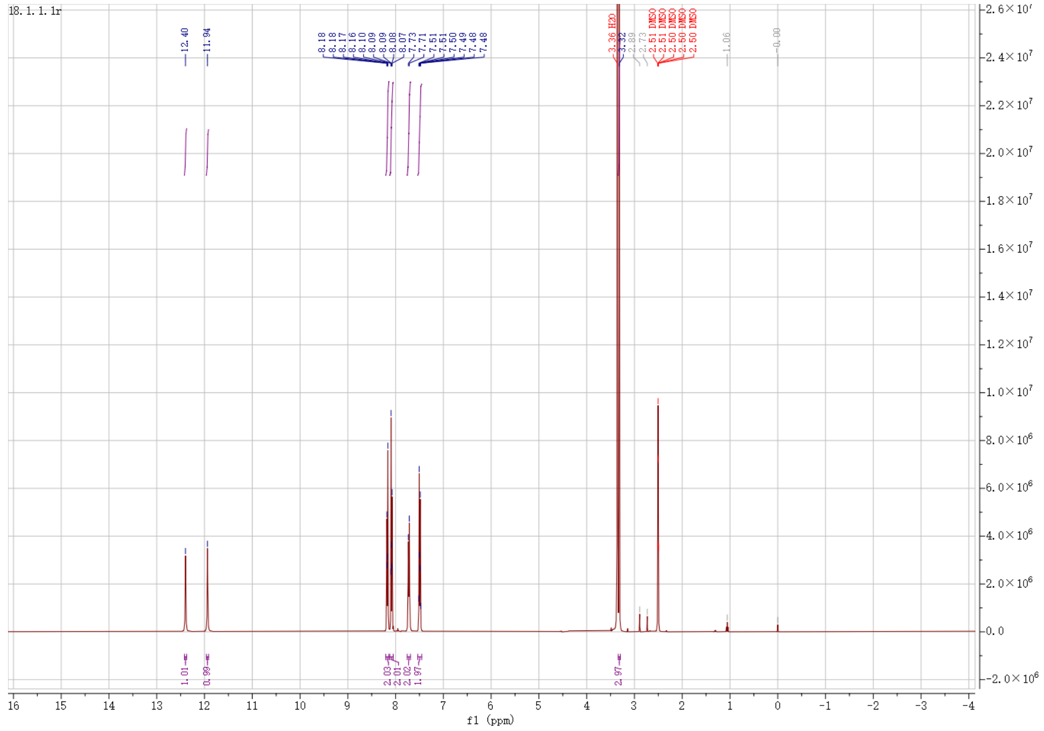

4r：N-(4-methylsulfonylbenzoyl)-N'-(2,4-dichlorophenyl) thiourea

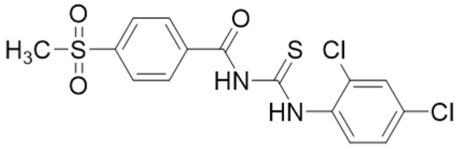

IR

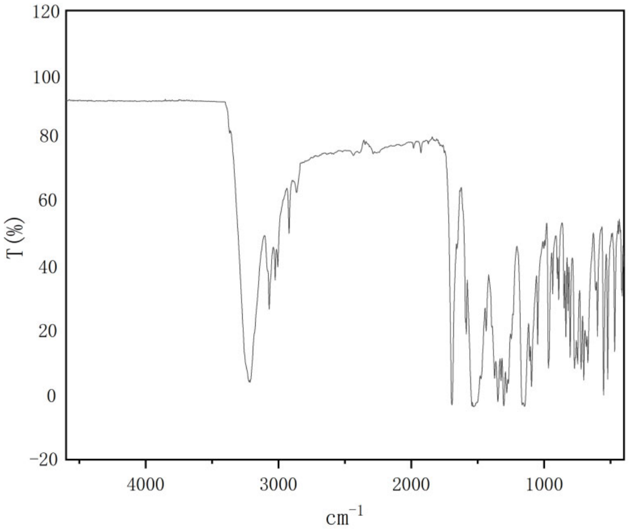

^{1}H NMR

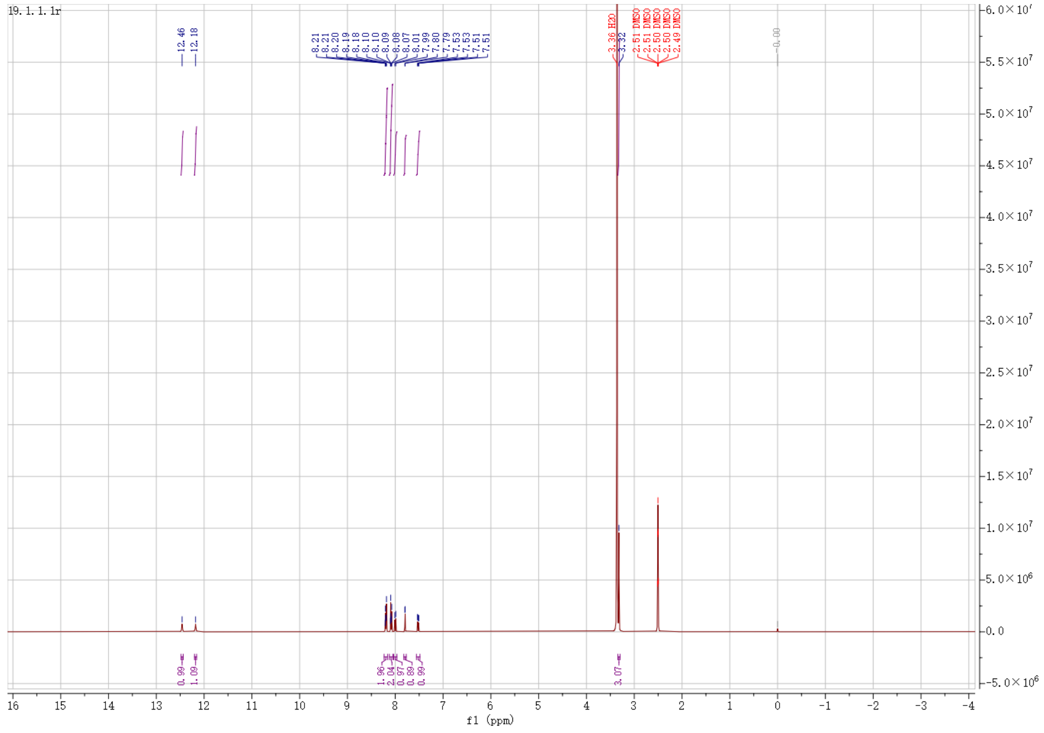

4s：N-(4-methylsulfonylbenzoyl)-N'-(4-methylphenyl) thiourea

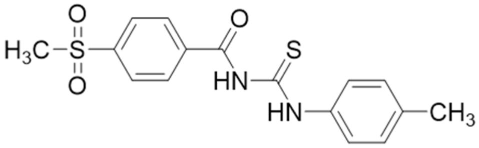

IR

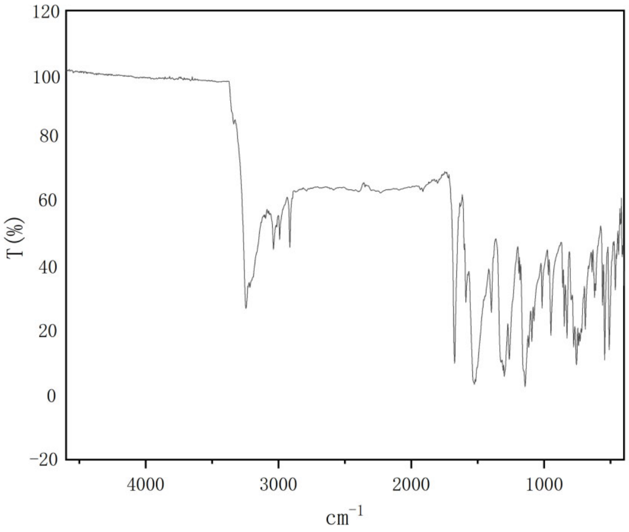

^{1}H NMR

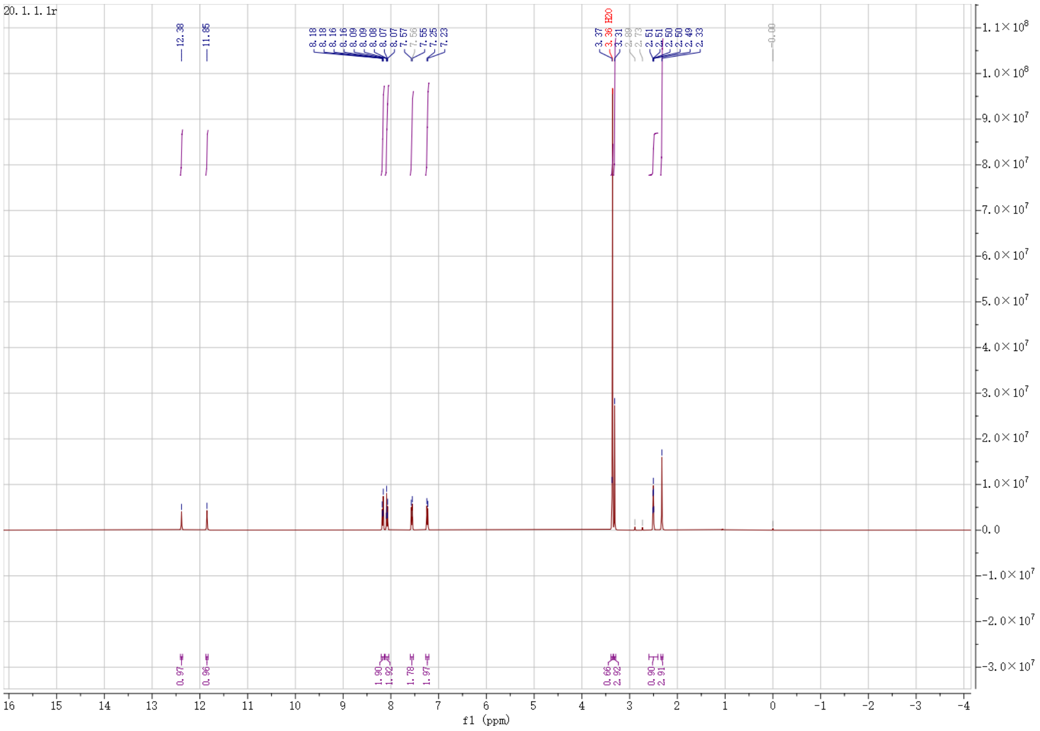

4t：N-(4-methylsulfonylbenzoyl)-N'-(3-methylphenyl) thiourea

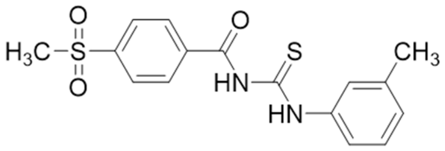

IR

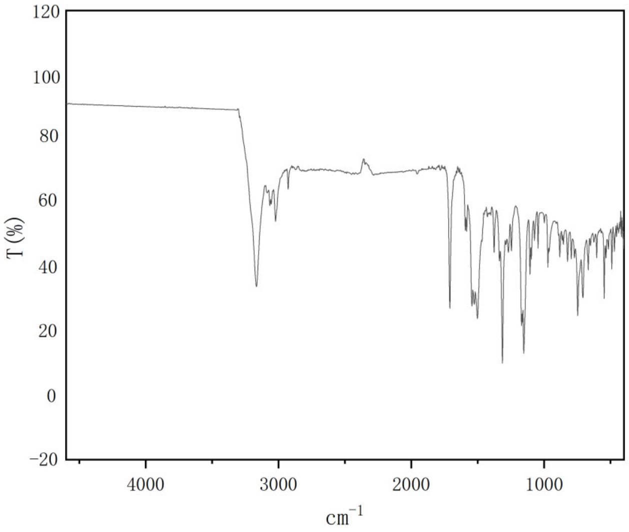

^{1}H NMR

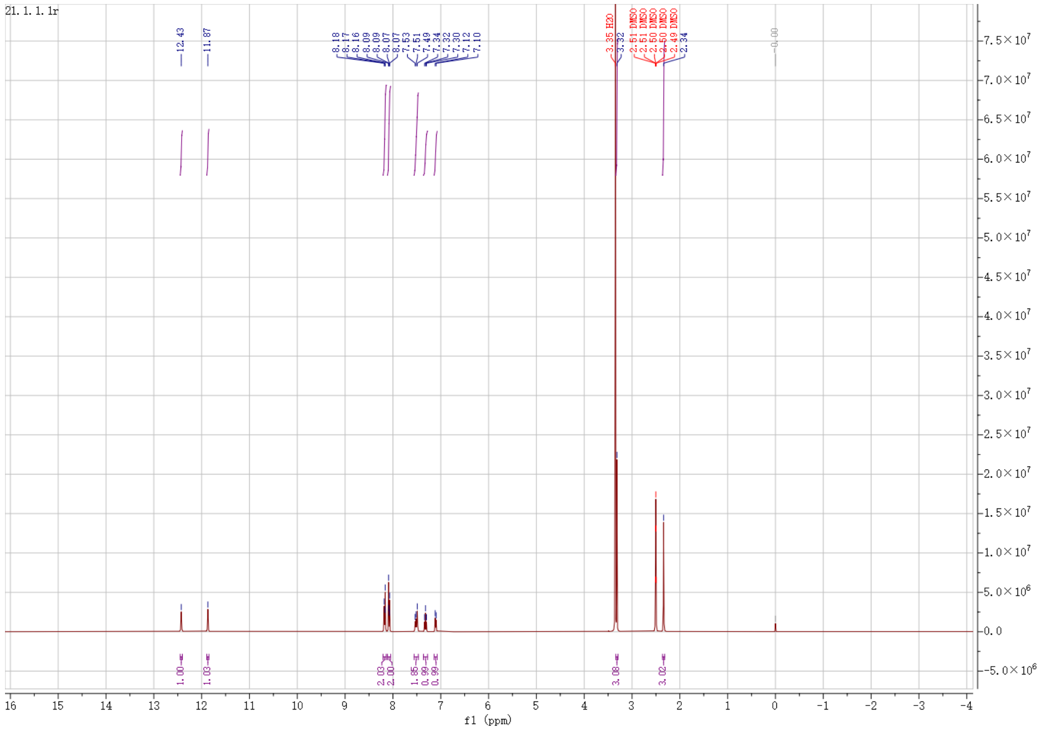

4u：N-(4-methylsulfonylbenzoyl)-N'-(2-nitrophenyl) thiourea

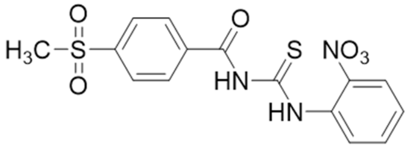

IR

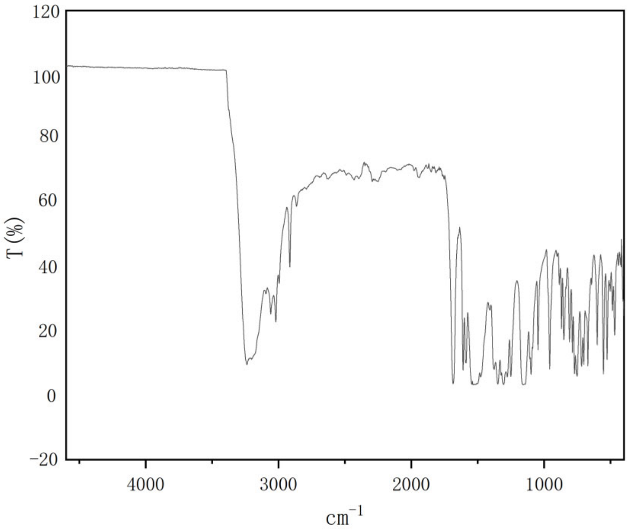

^{1}H NMR

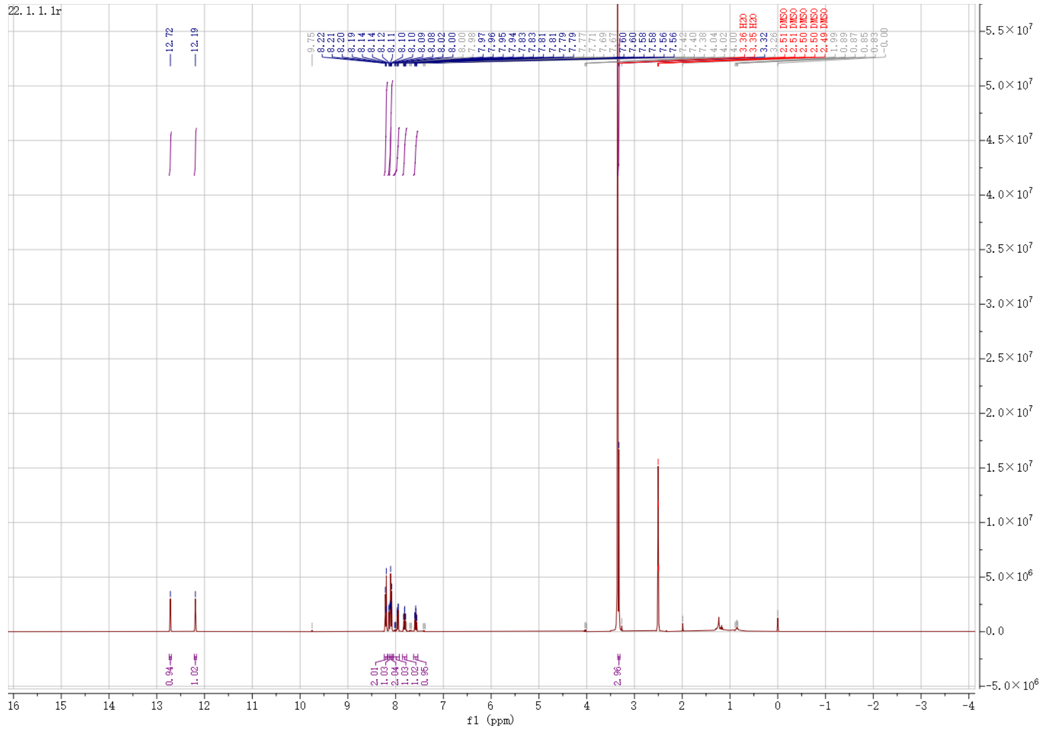

4v：N-(4-methylsulfonylbenzoyl)-N’-(phenyl) thiourea

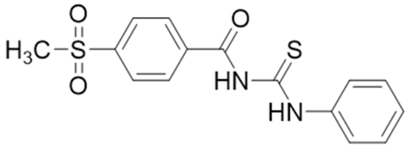

IR

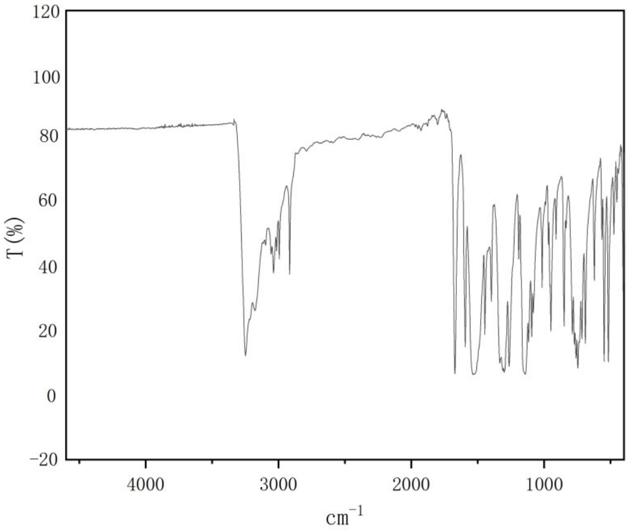

^{1}H NMR

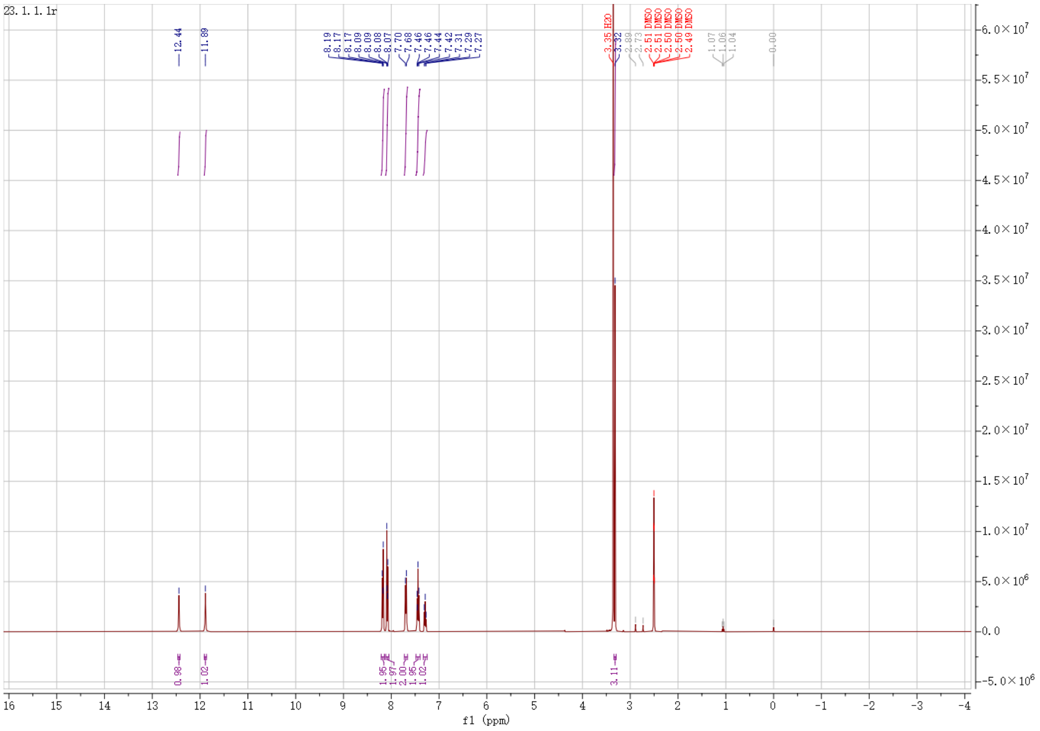

4w: N-(4-methylsulfonylbenzoyl)-N'-(2,4-dimethylphenyl) thiourea

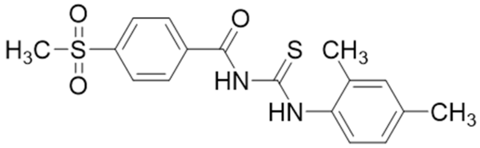

IR

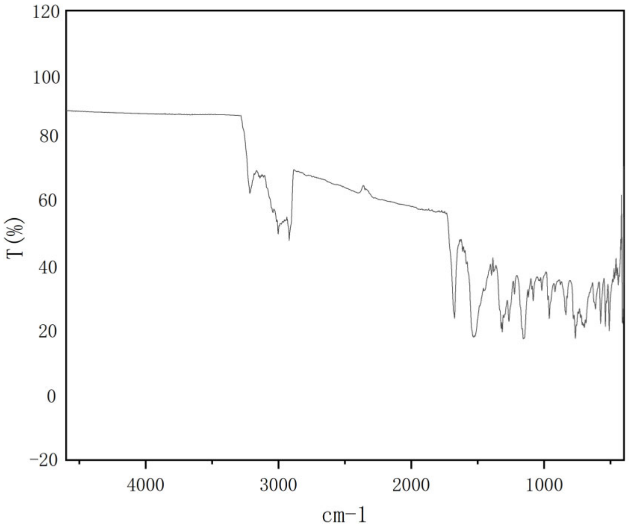

^{1}H NMR

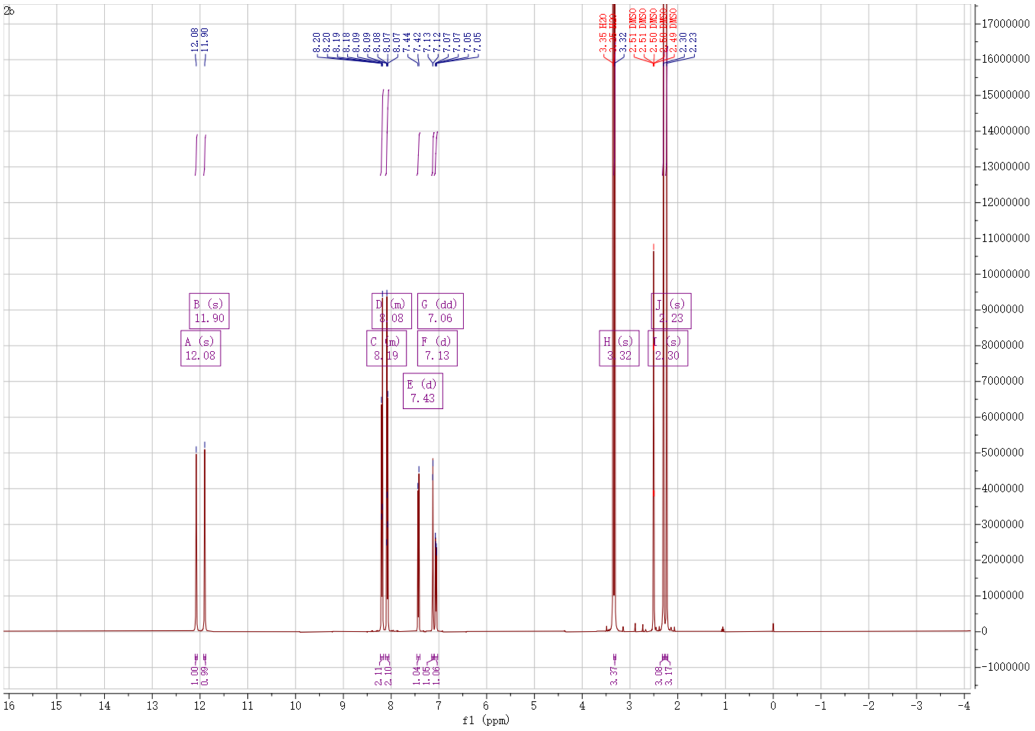

Supplement: Supplementary file 1 [file molecules-31-03162-s001.zip › molecules-4505444-supplementary.pdf]
